# Supplementary material for: Simultaneous estimation of bi-directional causal effects and heritable confounding from GWAS summary statistics
Source: Nat Commun. 2021 Dec 14;12:7274. doi: 10.1038/s41467-021-26970-w (PMC8671515; doi:10.1038/s41467-021-26970-w)
Supplement: Supplementary file 1 — Supplementary Information [file 41467_2021_26970_MOESM1_ESM.pdf]

Supplementary Information

Supplementary Methods

1.1 Characteristic functions of $z_k^{(u)}$, $z_k^{(x)}$, $z_k^{(y)}$ and (η_k^x, η_k^y)

The bivariate probability density function (PDF) of these summary statistics cannot be obtained analytically, but in the following we demonstrate that the characteristic function can be derived. Let us first compute the characteristic function of this two-dimensional random variable, knowing that $z_k^{(x)}, z_k^{(u)}, z_k^{(y)}$ and (η_k^x, η_k^y) are independent, hence the characteristic function can be factorised:

$$\begin{aligned}
\varphi_{\left(\widehat{\beta}_k^x, \widehat{\beta}_k^y\right)}(v,w) &= E\left[\exp\left(i \cdot \left(v \cdot \widehat{\beta}_k^x + w \cdot \widehat{\beta}_k^y\right)\right)\right] \\
&= E\left[\exp\left(i \cdot \left(v \cdot \left(\frac{z_k^{(x)} + (\alpha_{y\to x} \cdot q_y + q_x) \cdot z_k^{(u)} + \alpha_{y\to x} \cdot z_k^{(y)}}{1 - \alpha_{x\to y}\alpha_{y\to x}} + \eta_k^x\right) + \right.\right.\right. \\
&\left.\left.\left. + \; w \cdot \left(\frac{z_k^{(y)} + (\alpha_{x\to y} \cdot q_x + q_y) \cdot z_k^{(u)} + \alpha_{x\to y} \cdot z_k^{(x)}}{1 - \alpha_{x\to y}\alpha_{y\to x}} + \eta_k^y\right)\right)\right)\right] \\
&= E\left[\exp\left(i \cdot z_k^{(u)} \cdot \frac{v \cdot (\alpha_{y\to x} \cdot q_y + q_x) + w \cdot (\alpha_{x\to y} \cdot q_x + q_y)}{1 - \alpha_{x\to y}\alpha_{y\to x}}\right)\right] \\
&\times E\left[\exp\left(i \cdot z_k^{(x)} \cdot \frac{v + \alpha_{x\to y} \cdot w}{1 - \alpha_{x\to y}\alpha_{y\to x}}\right)\right] \cdot E\left[\exp\left(i \cdot z_k^{(y)} \cdot \frac{w + \alpha_{y\to x} \cdot v}{1 - \alpha_{x\to y}\alpha_{y\to x}}\right)\right] \\
&\times E\left[\exp\left(i \cdot \left(v \cdot \eta_k^x + w \cdot \eta_k^y\right)\right)\right] \\
&= \varphi_{z_k^{(u)}}\left(\frac{v \cdot (\alpha_{y\to x} \cdot q_y + q_x) + w \cdot (\alpha_{x\to y} \cdot q_x + q_y)}{1 - \alpha_{x\to y}\alpha_{y\to x}}\right) \\
&\times \varphi_{z_k^{(x)}}\left(\frac{v + \alpha_{x\to y} \cdot w}{1 - \alpha_{x\to y}\alpha_{y\to x}}\right) \cdot \varphi_{z_k^{(y)}}\left(\frac{w + \alpha_{y\to x} \cdot v}{1 - \alpha_{x\to y}\alpha_{y\to x}}\right) \cdot \varphi_{\left(\eta_k^x, \eta_k^y\right)}(v,w)
\end{aligned} \tag{1}$$

In the following we will work out each of the characteristic functions on the right hand side.

It is reasonable to assume that linkage disequilibrium (LD) fades off beyond 1Mb distance. Thus, without loss of generality we can assume that non-zero LD does not extend beyond m_0 markers around the focal variant. Hence we can assume that the length of $\boldsymbol{\rho}_k$ is m_0 and only consider $\boldsymbol{\gamma_x}, \boldsymbol{\gamma_y}$ and $\boldsymbol{\gamma_u}$ to be of length m_0 instead of m. Let us first approximate the distribution of $\boldsymbol{\rho}_k$ values following a spike and slab Gaussian mixture, i.e. proportion π_k of the m_0 SNPs have non-zero LD, coming from a Gaussian distribution $\mathcal{N}(0, \sigma_k^2)$ and the remaining $(1 - \pi_k)$ fraction of the LD values is zero. In mathematical notation

$$\boldsymbol{\rho}_k = \boldsymbol{r}_k \odot \boldsymbol{\kappa}_k \quad \text{with} \quad \boldsymbol{r_k} \sim \mathcal{N}(0, \sigma_k^2 \cdot I) \quad \text{and} \quad \boldsymbol{\kappa}_k \sim \mathcal{B}_{m_0}(1, \pi_k)$$

Therefore $z_k^{(u)}$ can be written of the form

$$\begin{aligned}
z_k^{(u)} &= \boldsymbol{\rho_k}' \cdot (\boldsymbol{\zeta_u} \odot \boldsymbol{\kappa_u}) = (\boldsymbol{r}_k \odot \boldsymbol{\kappa}_k)' \cdot (\boldsymbol{\zeta_u} \odot \boldsymbol{\kappa_u}) = (\boldsymbol{r}_k \odot \boldsymbol{\zeta_u})' \cdot (\underbrace{\boldsymbol{\kappa}_k \odot \boldsymbol{\kappa_u}}_{\boldsymbol{\kappa_{k,u}} \sim \mathcal{B}(1, \pi_k \cdot \pi_u)}) \\
&= \sum_{j=1}^{m_0} (\boldsymbol{r}_k \odot \boldsymbol{\zeta_u})_j \cdot (\boldsymbol{\kappa_{k,u}})_j
\end{aligned} \tag{2}$$

The PDF of the product of two zero-mean Gaussians ($\boldsymbol{r}_k$ and $\boldsymbol{\zeta_u}$) is a modified Bessel function of the second kind of order zero ($K_0(\omega)$) [1], more precisely

$$f_{(\boldsymbol{r}_k \odot \boldsymbol{\zeta_u})_j}(t) = \frac{1/\pi}{\sigma_u \cdot \sigma_k} \cdot K_0\left(\frac{|t|}{\sigma_u \cdot \sigma_k}\right) \tag{3}$$

and its characteristic function [2,3] is

$$\varphi_0(t) = E(\exp(i \cdot t \cdot (\boldsymbol{r}_k \odot \boldsymbol{\zeta_u})_j)) = \frac{1}{\sqrt{\sigma_u^2 \cdot \sigma_k^2 \cdot t^2 + 1}} \tag{4}$$

Next, the characteristic function of the product of $(\boldsymbol{r}_k \odot \boldsymbol{\zeta_u})_j$ and a Bernoulli distributed $(\boldsymbol{\kappa_{k,u}})_j$ is

$$\begin{aligned}\varphi_1(t) &= E\left(\exp\left(i \cdot t \cdot (\boldsymbol{r}_k \odot \boldsymbol{\zeta_u})_j\right) \cdot (\boldsymbol{\kappa_{k,u}})_j)\right) \\ &= \pi_k \cdot \pi_u \cdot E(\exp(i \cdot t \cdot (\boldsymbol{r}_k \odot \boldsymbol{\zeta_u})_j)) + (1 - \pi_k \cdot \pi_u) \cdot E(\exp(i \cdot t \cdot 0)) \\ &= \pi_k \cdot \pi_u \cdot \varphi_0(t) + (1 - \pi_k \cdot \pi_u) \\ &= \frac{\pi_k \cdot \pi_u}{\sqrt{\sigma_u^2 \cdot \sigma_k^2 \cdot t^2 + 1}} + (1 - \pi_k \cdot \pi_u)\end{aligned} \tag{5}$$

Hence the characteristic function of the sum of m_0 independent random variables is the product of them, we have

$$\varphi_{z_k^{(u)}}(t) = \left(\frac{\pi_k \cdot \pi_u}{\sqrt{\sigma_u^2 \cdot \sigma_k^2 \cdot t^2 + 1}} + (1 - \pi_k \cdot \pi_u)\right)^{m_0} \tag{6}$$

Finally, we apply a first order Taylor series approximation (around 1) of the log of the characteristic function in order to speed up computation and improve numerical accuracy

$$\begin{aligned}\log(\varphi_{z_k^{(u)}}(t)) &= m_0 \cdot \log\left(\frac{\pi_k \cdot \pi_u}{\sqrt{\sigma_u^2 \cdot \sigma_k^2 \cdot t^2 + 1}} + (1 - \pi_k \cdot \pi_u)\right) \\ &= m_0 \cdot \log\left(1 - \pi_k \cdot \pi_u \cdot \left(1 - \frac{1}{\sqrt{\sigma_u^2 \cdot \sigma_k^2 \cdot t^2 + 1}}\right)\right) \\ &\approx -m_0 \cdot \pi_k \cdot \pi_u \cdot \left(1 - \frac{1}{\sqrt{\sigma_u^2 \cdot \sigma_k^2 \cdot t^2 + 1}}\right)\end{aligned} \tag{7}$$

Analogously, the approximation of the logarithm of the characteristic functions of $z_k^{(x)}$ and $z_k^{(y)}$ is

$$\log(\varphi_{z_k^{(x)}}(t)) \approx -m_0 \cdot \pi_k \cdot \pi_x \cdot \left(1 - \frac{1}{\sqrt{\sigma_x^2 \cdot \sigma_k^2 \cdot t^2 + 1}}\right) \tag{8}$$

$$\log(\varphi_{z_k^{(y)}}(t)) \approx -m_0 \cdot \pi_k \cdot \pi_y \cdot \left(1 - \frac{1}{\sqrt{\sigma_y^2 \cdot \sigma_k^2 \cdot t^2 + 1}}\right) \tag{9}$$

Since the characteristic function of a centred multivariate Gaussian with variance-covariance matrix Σ is $\exp(-(1/2) \cdot \boldsymbol{t}' \cdot \Sigma \cdot \boldsymbol{t})$ we have

$$\log\left(\varphi_{(\eta_k^x, \eta_k^y)}(v, w)\right) = -\frac{1}{2} \cdot \left(\frac{i_x}{n_x} \cdot v^2 + 2 \cdot \frac{i_{x,y}}{\sqrt{n_x \cdot n_y}} \cdot v \cdot w + \frac{i_y}{n_y} \cdot w^2\right) \tag{10}$$

1.2 From characteristic function to probability density function

The final form of the logarithm of the joint characteristic function of the transformed summary statistics is

$$
\begin{aligned}
\log\left(\varphi_{\left(\widehat{\beta}_k^x,\widehat{\beta}_k^y\right)}(v,w)\right) &= \log\left(\varphi_{z_k^{(x)}}\left(\frac{v+\alpha_{x\to y}w}{1-\alpha_{x\to y}\alpha_{y\to x}}\right)\right) + \log\left(\varphi_{z_k^{(y)}}\left(\frac{w+\alpha_{y\to x}v}{1-\alpha_{x\to y}\alpha_{y\to x}}\right)\right) \\
&+ \log\left(\varphi_{z_k^{(u)}}\left(\frac{v\cdot(\alpha_{y\to x}\cdot q_y+q_x)+w\cdot(\alpha_{x\to y}\cdot q_x+q_y)}{1-\alpha_{x\to y}\alpha_{y\to x}}\right)\right) \\
&+ \log\left(\varphi_{\left(\eta_k^x,\eta_k^y\right)}(v,w)\right) \\
&\approx -m_0\cdot\pi_k\cdot\pi_x\cdot\left(1-\frac{1}{\sqrt{\frac{\sigma_x^2\cdot\sigma_k^2\cdot(v+\alpha_{x\to y}w)^2}{(1-\alpha_{x\to y}\alpha_{y\to x})^2}+1}}\right) \qquad (11) \\
&- m_0\cdot\pi_k\cdot\pi_y\cdot\left(1-\frac{1}{\sqrt{\frac{\sigma_y^2\cdot\sigma_k^2\cdot(w+\alpha_{y\to x}v)^2}{(1-\alpha_{x\to y}\alpha_{y\to x})^2}+1}}\right) \\
&- m_0\cdot\pi_k\cdot\pi_u\cdot\left(1-\frac{1}{\sqrt{\frac{\sigma_u^2\cdot\sigma_k^2\cdot(v\cdot(\alpha_{y\to x}\cdot q_y+q_x)+w\cdot(\alpha_{x\to y}\cdot q_x+q_y))^2}{(1-\alpha_{x\to y}\alpha_{y\to x})^2}+1}}\right) \\
&- \frac{1}{2}\cdot\left(\frac{i_x}{n_x}\cdot v^2+2\cdot\frac{i_{x,y}}{\sqrt{n_x\cdot n_y}}\cdot v\cdot w+\frac{i_y}{n_y}\cdot w^2\right)
\end{aligned}
$$

Using the inversion theorem for characteristic functions we can express the joint distribution of $\left(\widehat{\beta}_k^x,\widehat{\beta}_k^y\right)$ as

$$
f_{\left(\widehat{\beta}_k^x,\widehat{\beta}_k^y\right)}(x,y) = \left(\frac{1}{2\pi}\right)^2\cdot\int_{-\infty}^{\infty}\int_{-\infty}^{\infty}\exp(-i\cdot(x\cdot v+y\cdot w))\cdot\varphi_{\left(\widehat{\beta}_k^x,\widehat{\beta}_k^y\right)}(v,w)\;dv\;dw \qquad (12)
$$

This integral can be efficiently computed by Fast Fourier Transformation (FFT, see [4] and references within). To speed up computation, we bin SNPs according to their π_k and σ_k values (10×10 bins with equidistant centres) and for SNPs in the same bin the PDF function is evaluated over a fine grid ($2^7\times 2^7$ combinations) using the FFT.

Note that any derivative of the likelihood function can be readily calculated as a FFT of the derivative of the characteristic function, i.e.

$$
\frac{\partial}{\partial\theta}f_{\left(\widehat{\beta}_k^x,\widehat{\beta}_k^y\right)}(x,y) = \left(\frac{1}{2\pi}\right)^2\cdot\int_{-\infty}^{\infty}\int_{-\infty}^{\infty}\exp(-i\cdot(x\cdot v+y\cdot w))\cdot\frac{\partial}{\partial\theta}\varphi_{\left(\widehat{\beta}_k^x,\widehat{\beta}_k^y\right)}(v,w)\;dv\;dw \qquad (13)
$$

1.3 Computation of the LD scores

We first took 4,773,627 SNPs with info (imputation certainty measure) ≥ 0.99 present in the association summary files from the second round of GWAS by the Neale lab[5]. This set was restricted to 4,650,107 common, high-quality SNPs, defined as being present in both UK10K and UK Biobank, having MAF $> 1\%$ in both data sets, non-significant ($P_{diff} > 0.05$) allele frequency difference between UK Biobank and UK10K and residing outside the HLA region (chr6:28.5-33.5Mb). For these SNPs, LD scores and regression weights were computed based on

3,781 individuals from the UK10K study[6]. To estimate the local LD distribution for each SNP (k), characterised by π_k, σ_k^2, we fitted a two-component Gaussian mixture distribution to the observed local correlations (focal SNP $+/-$ 2'500 markers with MAF$\geq$ 0.5% in the UK10K): (1) one Gaussian component corresponding to zero correlations, reflecting only measurement noise (whose variance is proportional to the inverse of the reference panel size) and (2) a second component with zero mean and a larger variance than the first component (encompassing measurement noise plus non-zero LD).

1.4 Likelihood function identifiability

The likelihood function is symmetric around U, but for simplicity we will consider the general case where the variables of U and X are flipped, although the same can be said for the variables of U and Y. The likelihood function is partially identifiable such that there exists for any given model parameters, another model with different parameters but with the exact same likelihood function.

Proof: given that the SNPs effects between trait X and the confounder U are flipped, the new parameters follow the following structure:

$$\begin{aligned}
h'_x &= t_x + t_y \cdot \alpha_{y\to x} && (14)\\
h'_y &= h_y && (15)\\
\alpha'_{y\to x} &= \alpha_{y\to x} && (16)\\
\alpha'_{x\to y} &= \frac{q_x \cdot \alpha_{x\to y} + q_y}{q_x + q_y \cdot \alpha_{y\to x}}\\
&= \frac{q_x(\alpha_{x\to y} + \frac{q_y}{q_x})}{q_x(1 + \frac{q_y}{q_x} \cdot \alpha_{y\to x})}\\
&= \frac{\alpha_{x\to y} + \frac{q_y}{q_x}}{1 + \frac{q_y}{q_x} \cdot \alpha_{y\to x}} && (17)
\end{aligned}$$

through inverse transformation,

$$\alpha_{x\to y} = \frac{\alpha'_{x\to y} + \frac{q'_y}{q'_x}}{1 + \frac{q'_y}{q'_x} \cdot \alpha_{y\to x}} \tag{18}$$

Plugging in $\alpha'_{x\to y}$ in the above equation, and simplifying $\frac{ty}{tx}$ by w and $\frac{ty'}{tx'}$ by w' to get the confounding ratio:

$$\begin{aligned}
\alpha_{x\to y} &= \frac{\alpha'_{x\to y} + w'}{1 + w' \cdot \alpha_{y\to x}}\\
\alpha_{x\to y} + \alpha_{x\to y} \cdot w' \cdot \alpha_{y\to x} &= \alpha'_{x\to y} + w'\\
\alpha_{x\to y} - \alpha'_{x\to y} &= w' - \alpha_{x\to y} \cdot w' \cdot \alpha_{y\to x}\\
\alpha_{x\to y} - \alpha'_{x\to y} &= w'(1 - \alpha_{x\to y} \cdot \alpha_{y\to x})\\
w' &= \frac{\alpha_{x\to y} - \alpha'_{x\to y}}{1 - \alpha_{x\to y} \cdot \alpha_{y\to x}} && (19)
\end{aligned}$$

inserting the complete form of $\alpha'_{x\to y}$,

$$
\begin{aligned}
w' &= \frac{\alpha_{x\to y} - \frac{\alpha_{x\to y}+\frac{q_y}{q_x}}{1+\frac{q_y}{q_x}\cdot\alpha_{y\to x}}}{1-\alpha_{x\to y}\cdot\alpha_{y\to x}} \\
&= \frac{\alpha_{x\to y}(1+w\cdot\alpha_{y\to x}) - w - \alpha_{x\to y}}{(1-\alpha_{x\to y}\cdot\alpha_{y\to x})(1+w\cdot\alpha_{y\to x})} \\
&= \frac{\alpha_{x\to y}\cdot w\cdot\alpha_{y\to x} - w}{(1-\alpha_{x\to y}\cdot\alpha_{y\to x})(1+w\cdot\alpha_{y\to x})} \\
&= \frac{w(\alpha_{x\to y}\cdot\alpha_{y\to x} - 1)}{(1-\alpha_{x\to y}\cdot\alpha_{y\to x})(1+w\cdot\alpha_{y\to x})} \\
&= \frac{-w}{1+w\cdot\alpha_{y\to x}} \qquad (20)
\end{aligned}
$$

In order to obtain t'_y and t'_x, we use the equations of h'_x, $\alpha'_{x\to y}$ and by using the inverse transformation of $\alpha'_{y\to x} = \alpha_{y\to x}$, $\alpha_{x\to y}$ as well as w' as follows:

$$
\begin{aligned}
t'_y &= \frac{-t'_x\cdot w}{1+w\cdot\alpha_{y\to x}} \qquad (21) \\
h_x &= t'_x + t'_y\cdot\alpha_{y\to x} \\
&= t'_x + \frac{-t'_x\cdot w}{1+w\cdot\alpha_{y\to x}}\cdot\alpha_{y\to x} \\
&= \frac{t'_x + t'_x\cdot w\cdot\alpha_{y\to x} - t'_x\cdot w\cdot\alpha_{y\to x}}{1+w\cdot\alpha_{y\to x}} \\
&= \frac{t'_x}{1+w\cdot\alpha_{y\to x}} \qquad (22) \\
t'_x &= h_x(1+w\cdot\alpha_{y\to x}) \qquad (23)
\end{aligned}
$$

Replacing t'_x in h_x to get t'_y:

$$
t'_y = h_x\cdot w \qquad (24)
$$

Under these two models with equal likelihood, there are three slopes obtained from the observed data: two are the correlation of effect sizes ($\alpha_{x\to y}$ and $1/\alpha_{y\to x}$), where one of them is greater than, and the other is within the parameter bounds. The third is the correlation of the confounder $\frac{\alpha_{x\to y}+\frac{q_y}{q_x}}{1+\frac{q_y}{q_x}\cdot\alpha_{y\to x}}$.

More often than not, only one slope is recovered within the boundaries of the parameters set for LHC-MR. However, given the now known re-parameterisation, the second (and if found, third) slope can be simply calculated if not found by the likelihood function minimisation. It is reasonable to assume that the direct heritability of each trait is larger than the indirect heritability, hence we report parameter sets where $h_x^2 > t_x^2$ or $h_y^2 > t_y^2$.

1.5 Decomposition of genetic correlation

Given the starting equations for X and Y we can calculate their genetic correlation. Denoting the total (multivariate) genetic effect for X and Y as $\boldsymbol{\delta_x}$ and $\boldsymbol{\delta_y}$, we can express them as follows

$$\boldsymbol{\delta_x} = q_x \cdot \boldsymbol{\gamma_u} + \alpha_{y\to x}\boldsymbol{\delta_y} + \boldsymbol{\gamma_x} \tag{25}$$

$$\boldsymbol{\delta_y} = q_y \cdot \boldsymbol{\gamma_u} + \alpha_{x\to y}\boldsymbol{\delta_x} + \boldsymbol{\gamma_y} \tag{26}$$

Substituting the second equation to the first yields

$$\begin{aligned}\boldsymbol{\delta_x} &= q_x \cdot \boldsymbol{\gamma_u} + \alpha_{y\to x}(q_y \cdot \boldsymbol{\gamma_u} + \alpha_{x\to y}\boldsymbol{\delta_x} + \boldsymbol{\gamma_y}) + \boldsymbol{\gamma_x} \\ &= (q_x + \alpha_{y\to x}q_y) \cdot \boldsymbol{\gamma_u} + (\alpha_{y\to x}\alpha_{x\to y})\boldsymbol{\delta_x} + \alpha_{y\to x}\boldsymbol{\gamma_y} + \boldsymbol{\gamma_x} \\ &= \left((q_x + \alpha_{y\to x}q_y) \cdot \boldsymbol{\gamma_u} + \alpha_{y\to x}\boldsymbol{\gamma_y} + \boldsymbol{\gamma_x}\right)/(1 - \alpha_{y\to x}\alpha_{x\to y})\end{aligned} \tag{27}$$

Similarly,

$$\boldsymbol{\delta_y} = \left((q_y + \alpha_{x\to y}q_x) \cdot \boldsymbol{\gamma_u} + \alpha_{x\to y}\boldsymbol{\gamma_x} + \boldsymbol{\gamma_y}\right)/(1 - \alpha_{y\to x}\alpha_{x\to y}) \tag{28}$$

Thus the genetic covariance is

$$\begin{aligned}E[\boldsymbol{\delta_x} \cdot \boldsymbol{\delta_y}] &= \left((q_x + \alpha_{y\to x}q_y) \cdot \boldsymbol{\gamma_u} + \alpha_{y\to x}\boldsymbol{\gamma_y} + \boldsymbol{\gamma_x}\right)\left((q_y + \alpha_{x\to y}q_x) \cdot \boldsymbol{\gamma_u} + \alpha_{x\to y}\boldsymbol{\gamma_x} + \boldsymbol{\gamma_y}\right)/(1 - \alpha_{y\to x}\alpha_{x\to y})^2 \\ &= \left((q_x + \alpha_{y\to x}q_y)(q_y + \alpha_{x\to y}q_x)h_u^2 + \alpha_{y\to x}h_y^2 + \alpha_{x\to y}h_x^2\right)/(1 - \alpha_{y\to x}\alpha_{x\to y})^2 \\ &= \left((t_x + \alpha_{y\to x}t_y)(t_y + \alpha_{x\to y}t_x) + \alpha_{y\to x}h_y^2 + \alpha_{x\to y}h_x^2\right)/(1 - \alpha_{y\to x}\alpha_{x\to y})^2\end{aligned} \tag{29}$$

and the heritabilities are

$$E[\boldsymbol{\delta_x^2}] = \left((t_x + \alpha_{y\to x}t_y)^2 + \alpha_{y\to x}^2h_y^2 + h_x^2\right)/(1 - \alpha_{y\to x}\alpha_{x\to y})^2 \tag{30}$$

$$E[\boldsymbol{\delta_y^2}] = \left((t_y + \alpha_{x\to y}t_x)^2 + \alpha_{x\to y}^2h_x^2 + h_y^2\right)/(1 - \alpha_{y\to x}\alpha_{x\to y})^2 \tag{31}$$

Therefore the genetic correlation takes the form

$$corr(\boldsymbol{\delta_x}, \boldsymbol{\delta_y}) = \frac{(t_x + \alpha_{y\to x}t_y)(t_y + \alpha_{x\to y}t_x) + \alpha_{y\to x}h_y^2 + \alpha_{x\to y}h_x^2}{\sqrt{\left((t_x + \alpha_{y\to x}t_y)^2 + \alpha_{y\to x}^2h_y^2 + h_x^2\right)\left((t_y + \alpha_{x\to y}t_x)^2 + \alpha_{x\to y}^2h_x^2 + h_y^2\right)}} \tag{32}$$

These values can be compared to those obtained by LD score regression.

References

[1] Nadarajah, S. and Pogány, T. K. (2016). On the distribution of the product of correlated normal random variables. Comptes Rendus Mathematique *354*, 201–204.

[2] McNolty, F. (1973). Some probability density functions and their characteristic functions. Mathematics of computation 27, 495-504.

[3] Bateman, H. (1953). Higher Transcendental Functions, Volume I. `https://authors.library.caltech.edu/43491/`.

[4] Heideman, M. T., Johnson, D. H., and Burrus, C. S. (1985). Gauss and the history of the fast fourier transform. Archive for History of Exact Sciences *34*, 265-277.

[5] Neale Lab (2018). UK BioBank. `http://www.nealelab.is/uk-biobank/`.

[6] Walter, K., Min, J. L., Huang, J., Crooks, L., Memari, Y., McCarthy, S., Perry, J. R. B., Xu, C., Futema, M., Lawson, D., et al. (2015). The uk10k project identifies rare variants in health and disease. Nature *526*, 82–90.

Supplementary Figures

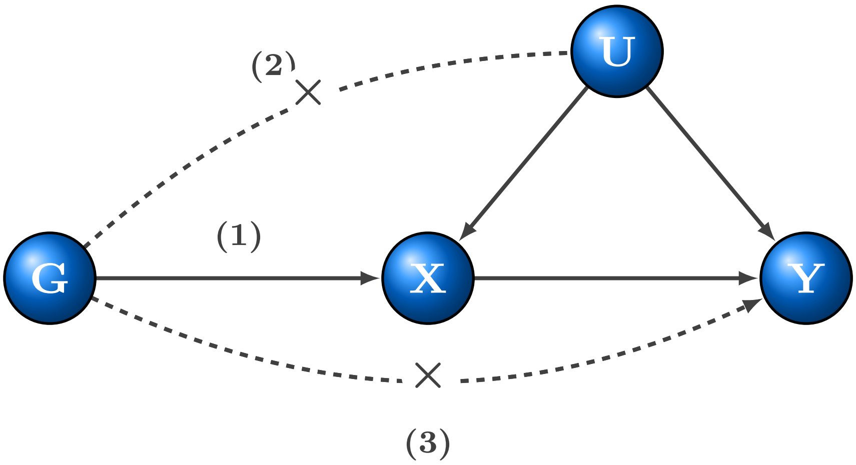

Supplementary Figure 1: Basic assumptions of Mendelian randomisation. (1) Relevance – genetic data, denoted by G, is robustly associated with the exposure. (2) Exchangeability – G is not associated with any confounder of the exposure-outcome relationship. (3) Exclusion restriction – G is independent of the outcome conditional on the exposure and all confounders of the exposure-outcome relationship (i.e. the only path between the instrument and the outcome is via the exposure).

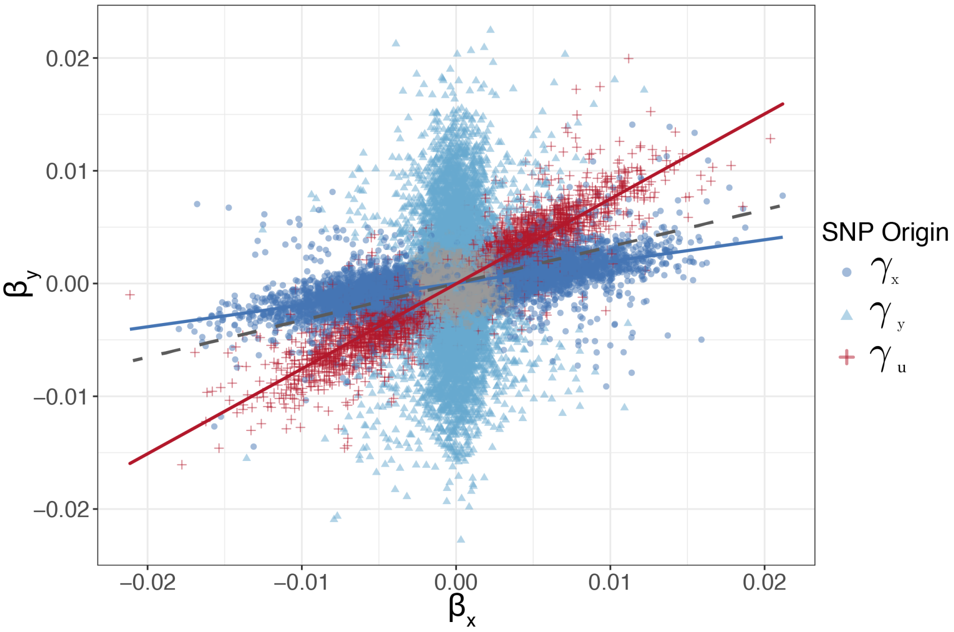

Supplementary Figure 2: An illustration of a scatter plot showing simulated observed SNP effects on traits X and Y, coloured by the strongest effect between the three vectors $\gamma_x, \gamma_y, \gamma_u$. SNPs in grey are those with no effect on any of the traits. This illustration shows the distinct clusters that could arise in the presence of a confounder. The dark blue cluster of SNPs represents those that are not in violation of any of the MR assumption, and hence its slope reflects the true causal effect of X on Y, while the red cluster of SNPs are those associated with the confounder. The steeper slope of the red cluster of SNPs causes a typical regression line - shown in grey - that represents the causal effect (estimated using conventional MR methods) to be overestimated.

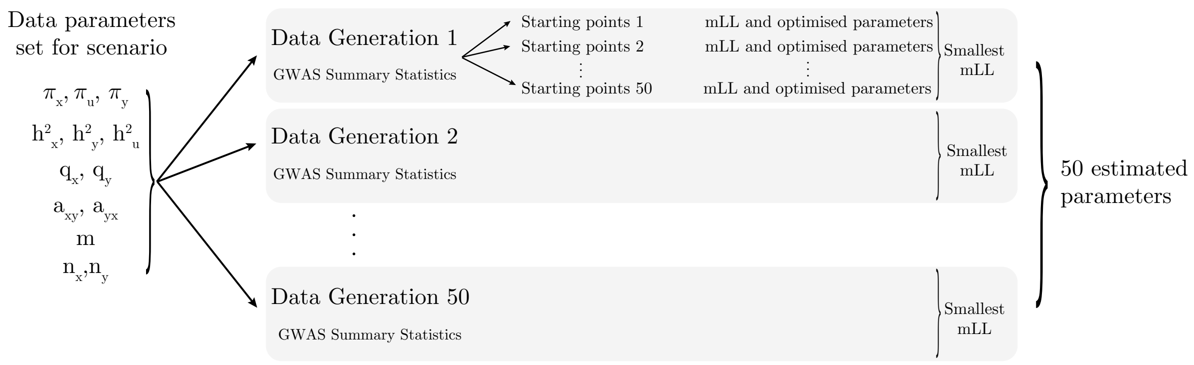

Supplementary Figure 3: A schema showing the workflow of the simulation results. For a single set of parameter settings, 50 different data generations of GWAS summary statistics are created for trait X and Y. The summary statistics of a single data generation, as well as the sample size, SNP number and SNP-based LD structure are used in the likelihood optimisation function that is run with 100 different random starting points in order to explore the likelihood surface. A single maximum likelihood and its corresponding estimated parameters are selected to represent the estimates of that data generation. And this is repeated for the other generations. The results for several data generation are often represented in boxplots throughout the paper.

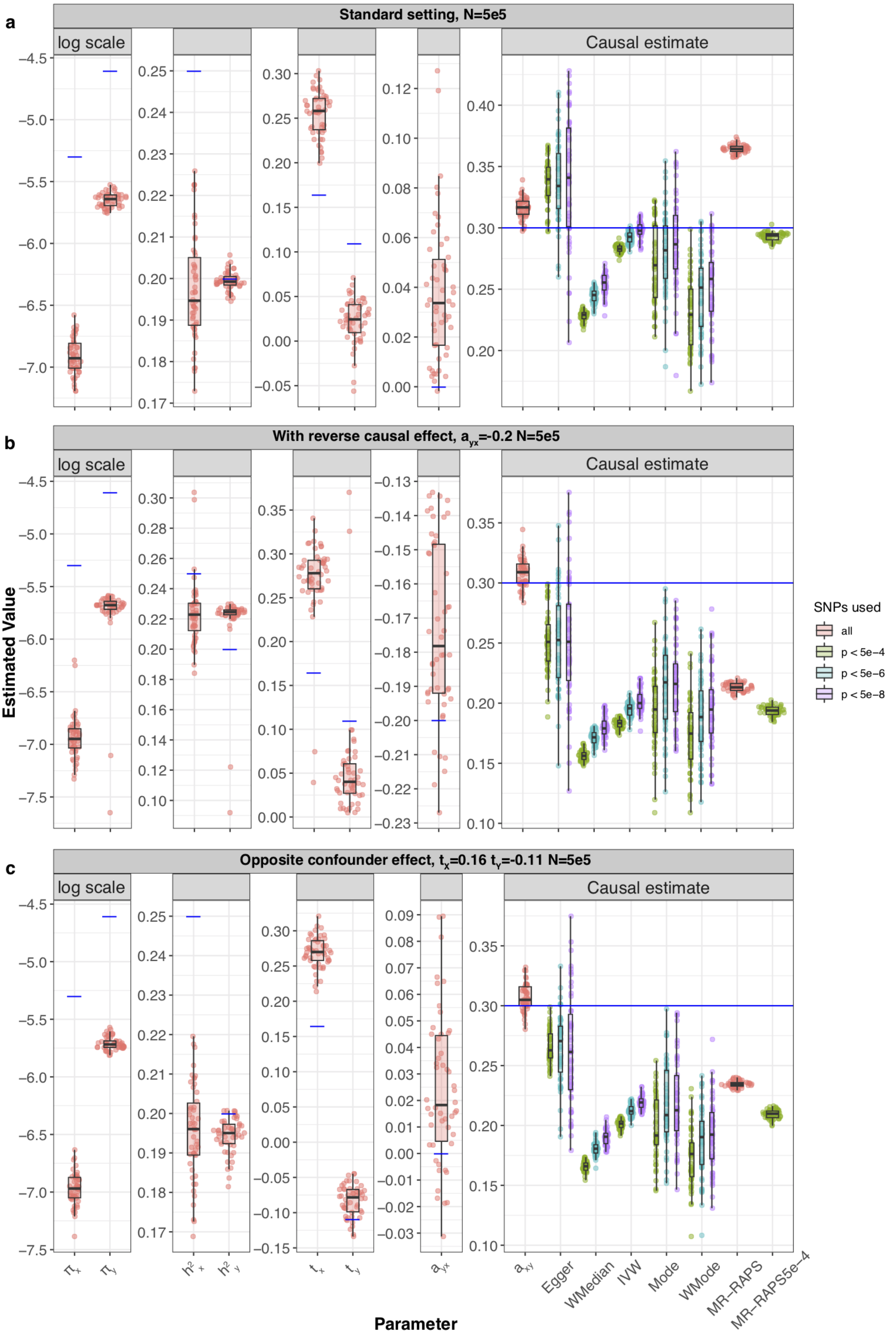

Supplementary Figure 4: Simulation results under various scenarios. These modified Sina-boxplot represent the distribution of parameter estimates from 50 different data generations under various conditions. For each generation, standard MR methods as well as our LHC-MR were used to estimate a causal effect. In the boxplots, the lower and upper hinges correspond to the first and third quartiles, the middle bar corresponds to the median, whereas the upper whisker is the largest dataset estimate smaller than 1.5∗inter-quartile range above the third quartile. The lower whisker is defined analogously. The true values of the parameters used in the data generations are represented by the blue dots/lines. **a** Estimation under standard settings ($\pi_x = 5 \times 10^{-3}, \pi_y = 1 \times 10^{-2}, \pi_u = 5 \times 10^{-2}, h_x^2 = 0.25, h_y^2 = 0.2, h_u^2 = 0.3, t_x = 0.16, t_y = 0.11$). **b** Addition of a reverse causal effect $\alpha_{y \to x} = -0.2$. **c** Confounder with opposite causal effects on X and Y ($t_x = 0.16, t_y = -0.11$).

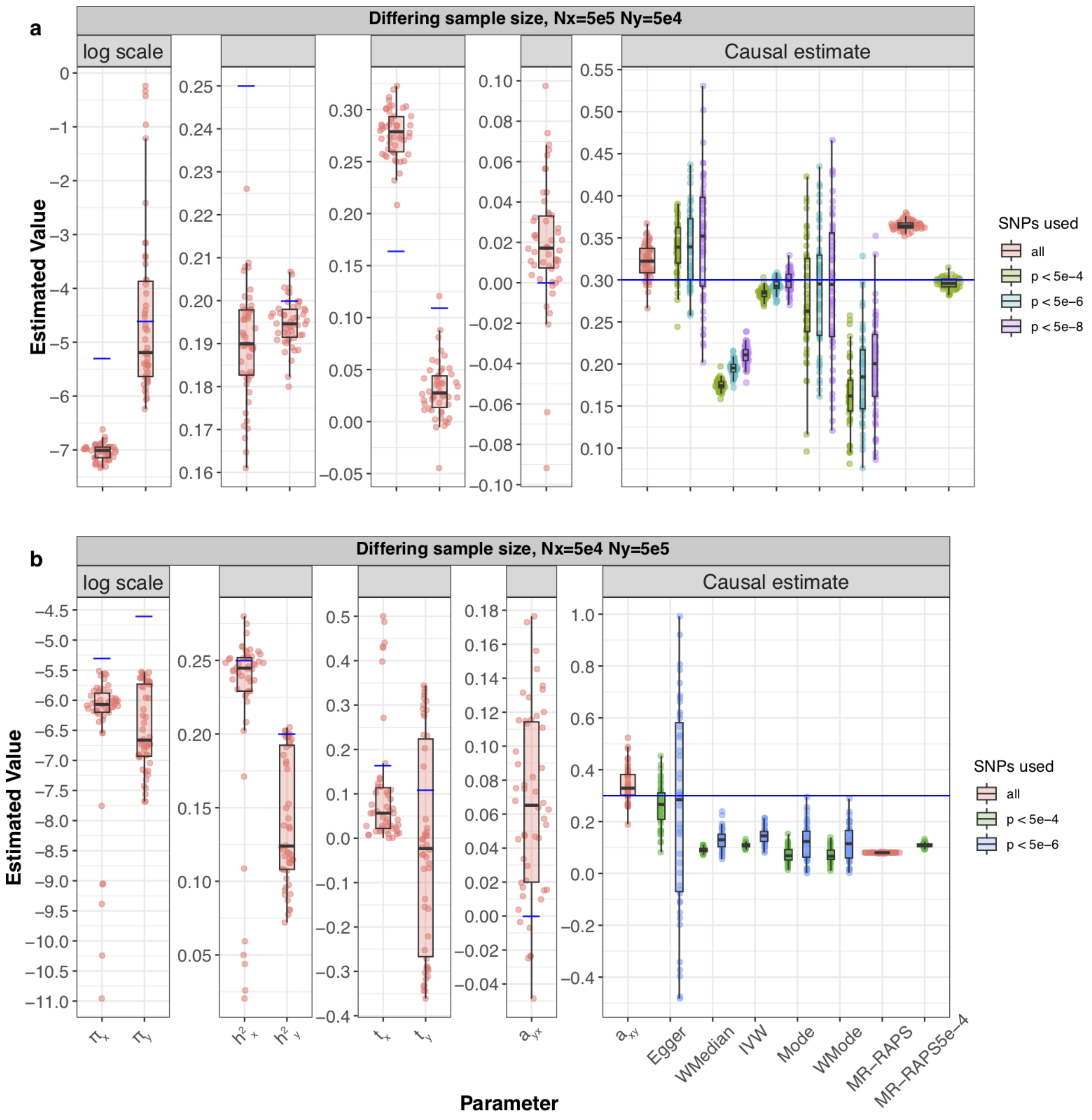

Supplementary Figure 5: Simulation results showing varying sample sizes for the two exposure and outcome samples. Modified Sina-boxplots representing the distribution of parameter estimates from 50 different data generations. For each generation, standard MR methods as well as our LHC-MR were used to estimate a causal effect. In the boxplots, the lower and upper hinges correspond to the first and third quartiles, the middle bar corresponds to the median, whereas the upper whisker is the largest dataset estimate smaller than 1.5∗inter-quartile range above the third quartile. The lower whisker is defined analogously. The true values of the parameters used in the data generations are represented by the blue dots/lines. In this figure, samples sizes for the two traits differ as such n_x = 500,000 and n_y = 50,000 for **a**, and n_x = 50,000 and n_y = 500,000 for **b**.

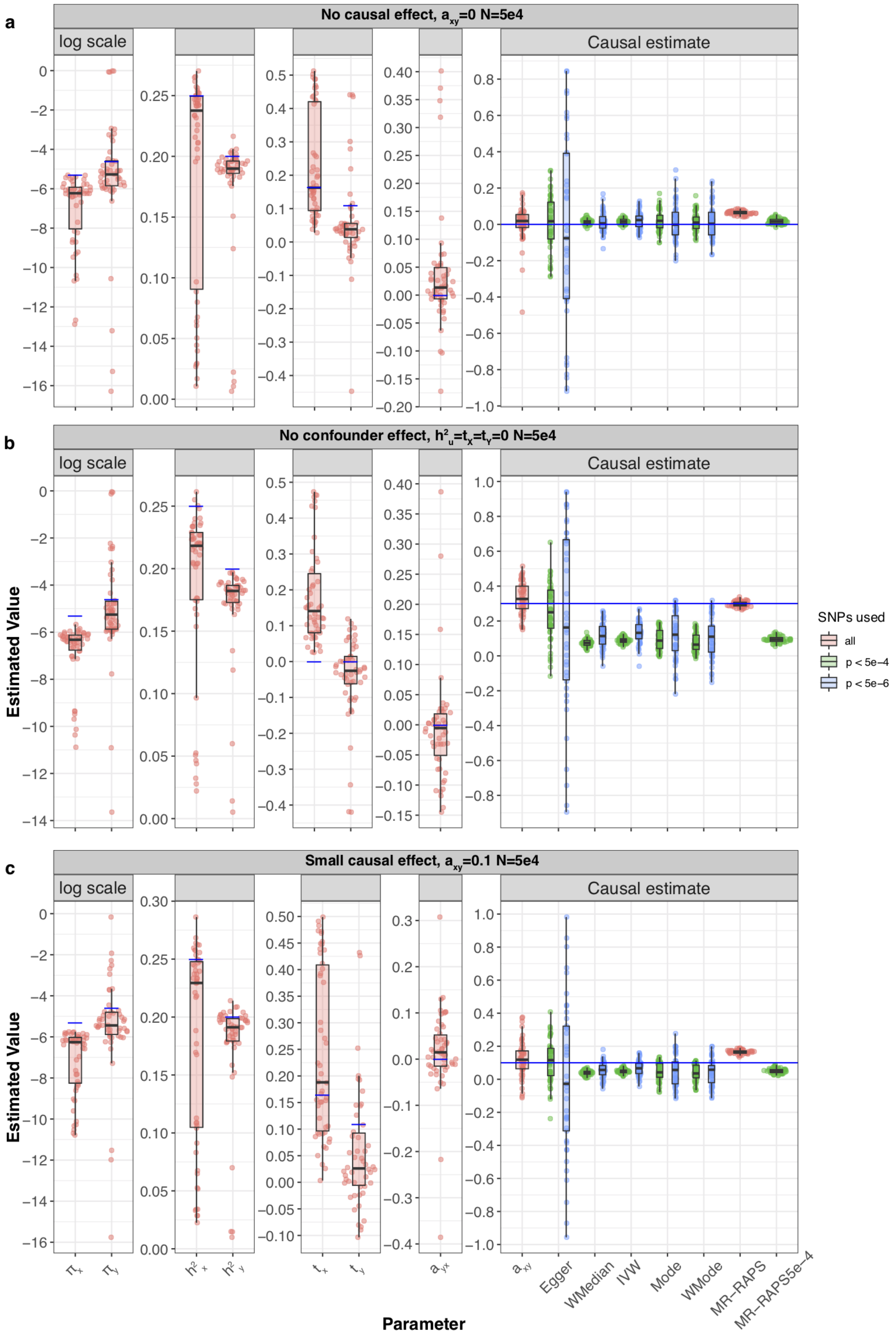

Supplementary Figure 6: Simulation results under various scenarios. These modified Sina-boxplots represent the distribution of parameter estimates from 50 different data generations under various conditions. For each generation, standard MR methods as well as our LHC-MR were used to estimate a causal effect. In the boxplots, the lower and upper hinges correspond to the first and third quartiles, the middle bar corresponds to the median, whereas the upper whisker is the largest dataset estimate smaller than 1.5∗inter-quartile range above the third quartile. The lower whisker is defined analogously. The true values of the parameters used in the data generations are represented by the blue dots/lines. **a** The data simulated had no causal effect in either direction. **b** The data simulated had no confounder effect with π_u, t_x, and $t_y = 0$. **c** This model had a small causal effect of $\alpha_{x \to y} = 0.1$.

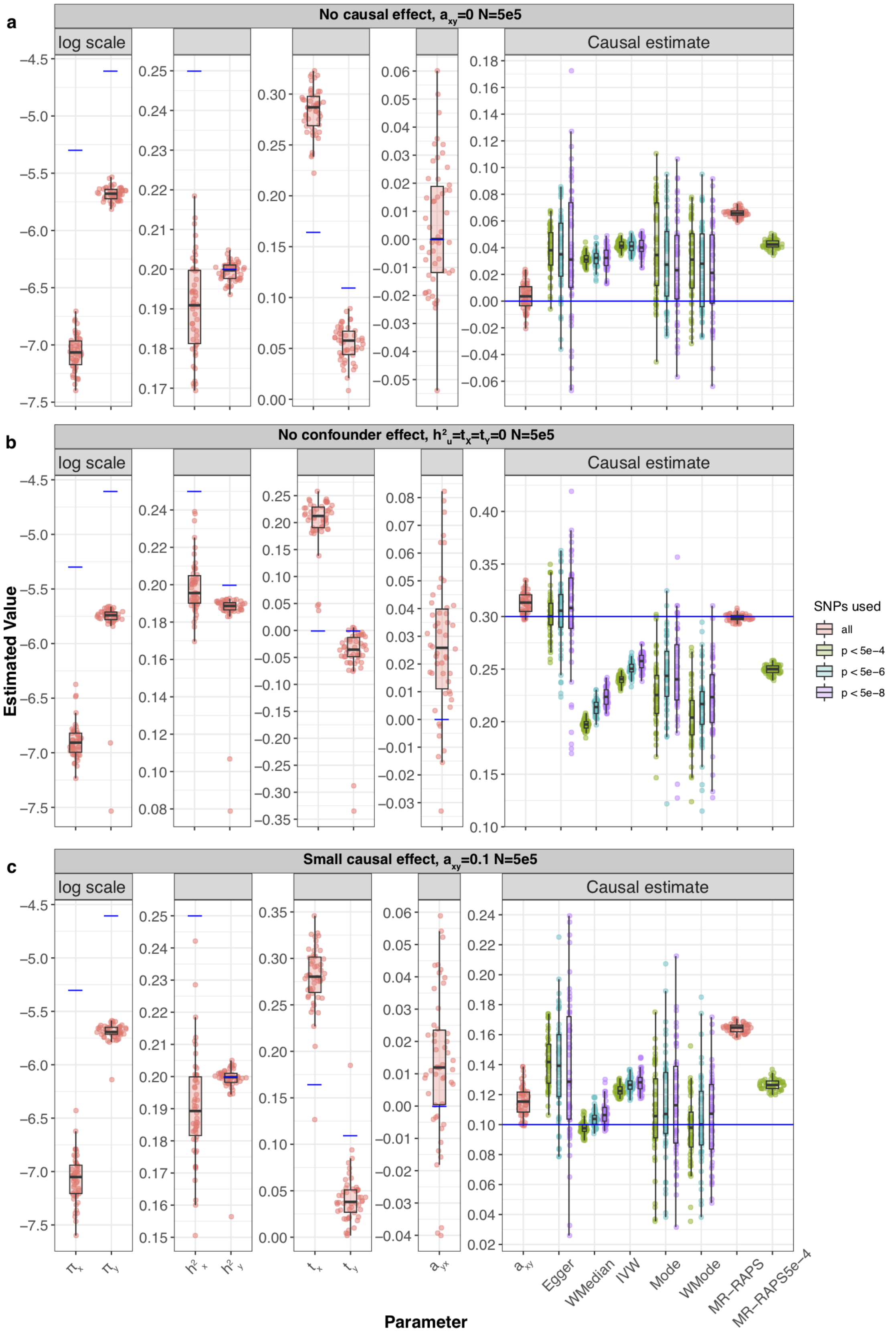

Supplementary Figure 7: **Simulation results under various scenarios.** These modified Sina-boxplots represent the distribution of parameter estimates from 50 different data generations under various conditions. For each generation, standard MR methods as well as our LHC-MR were used to estimate a causal effect. In the boxplots, the lower and upper hinges correspond to the first and third quartiles, the middle bar corresponds to the median, whereas the upper whisker is the largest dataset estimate smaller than 1.5∗inter-quartile range above the third quartile. The lower whisker is defined analogously. The true values of the parameters used in the data generations are represented by the blue dots/lines. **a** The data simulated had no causal effect in either direction. **b** The data simulated had no confounder effect with π_u, t_x, and $t_y = 0$. **c** This model had a small causal effect of $\alpha_{x\rightarrow y} = 0.1$.

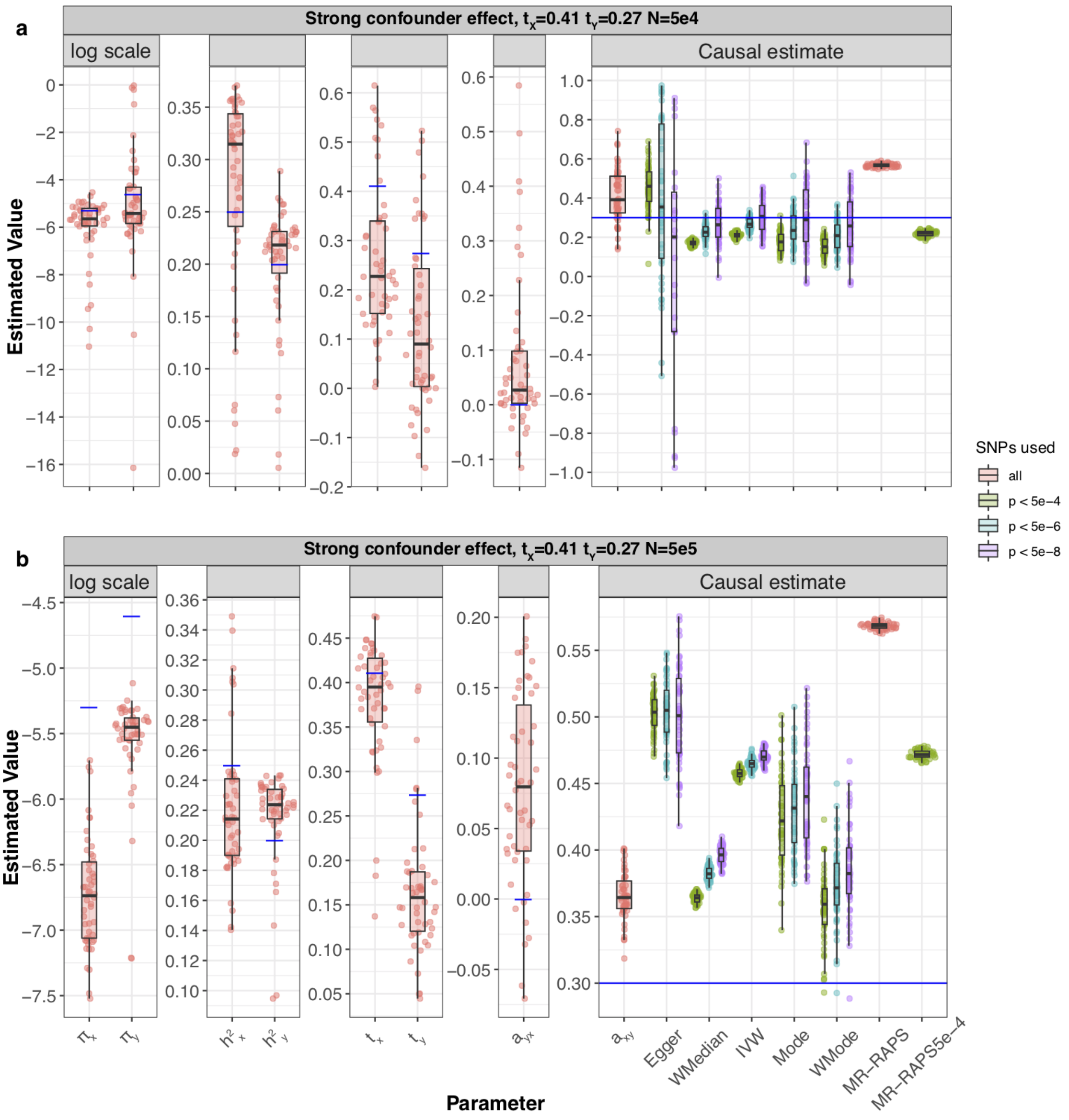

Supplementary Figure 8: Simulation results under various scenarios. These modified Sina-boxplots represent the distribution of parameter estimates from 50 different data generations under various conditions. For each generation, standard MR methods as well as our LHC-MR were used to estimate a causal effect. In the boxplots, the lower and upper hinges correspond to the first and third quartiles, the middle bar corresponds to the median, whereas the upper whisker is the largest dataset estimate smaller than 1.5∗inter-quartile range above the third quartile. The lower whisker is defined analogously. The true values of the parameters used in the data generations are represented by the blue dots/lines. **a** The data simulated shows the increased effect of U on X and Y through $t_x = 0.41, t_y = 0.27$ instead of the standard setting $t_x = 0.16, t_y = 0.11$. **b** This panel show the same thing but with a larger sample size of $n_x = n_y = 500,000$

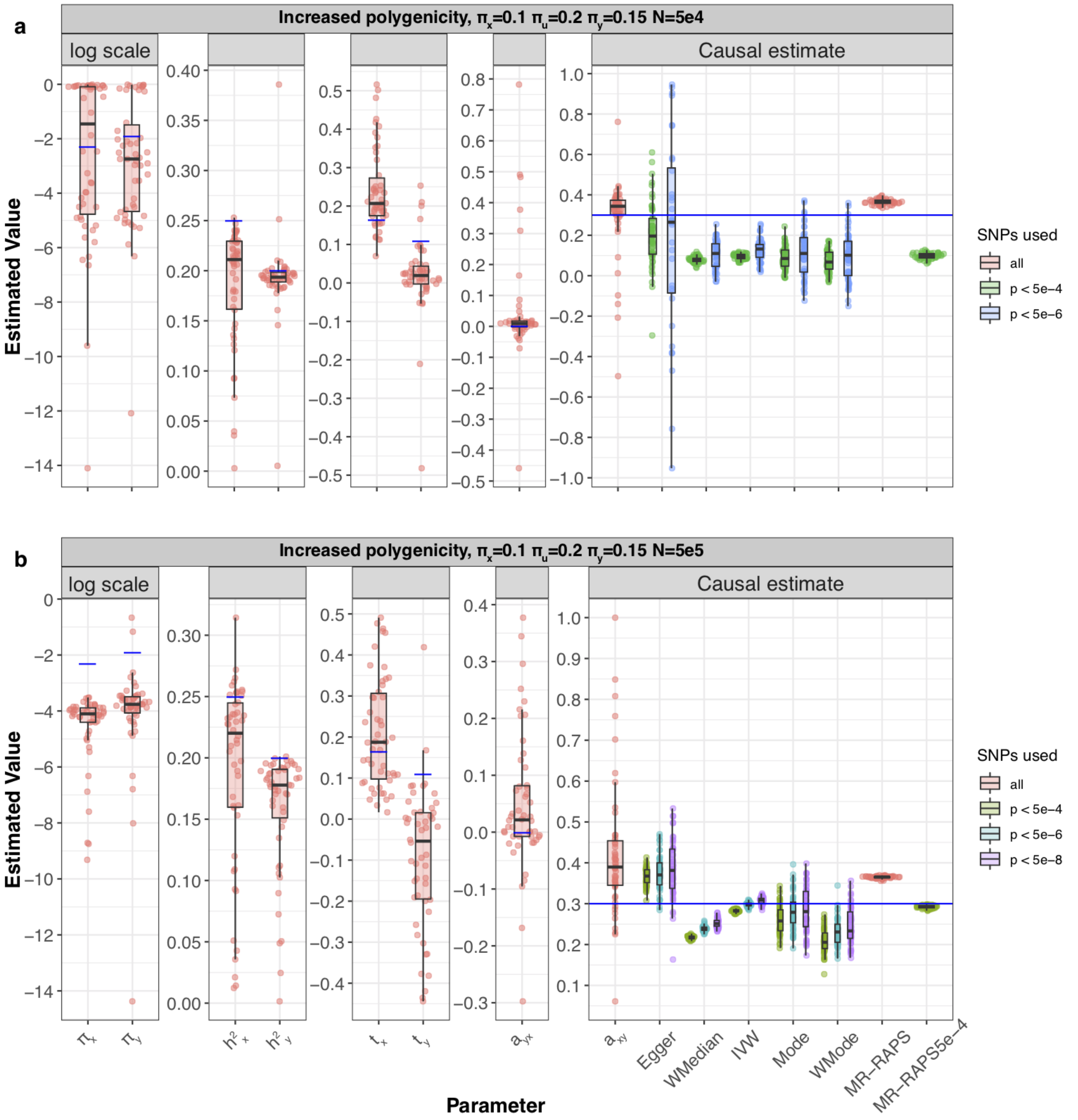

Supplementary Figure 9: Simulation results where there is an increased polygenicity for all traits. Modified Sina-boxplots representing the distribution of parameter estimates from 50 different data generations. For each generation, standard MR methods as well as our LHC-MR were used to estimate a causal effect. In the boxplots, the lower and upper hinges correspond to the first and third quartiles, the middle bar corresponds to the median, whereas the upper whisker is the largest dataset estimate smaller than 1.5∗inter-quartile range above the third quartile. The lower whisker is defined analogously. The true values of the parameters used in the data generations are represented by the blue dots/lines. The proportion of effective SNPs that make up the spike-and-slab distributions of the γ vectors in this setting is $10\%, 15\%, and 20\%$ for traits X, Y and U respectively. **a** Results for smaller sample size of $n_x = n_y = 50,000$. **b** Results for larger sample size of $n_x = n_y = 500,000$.

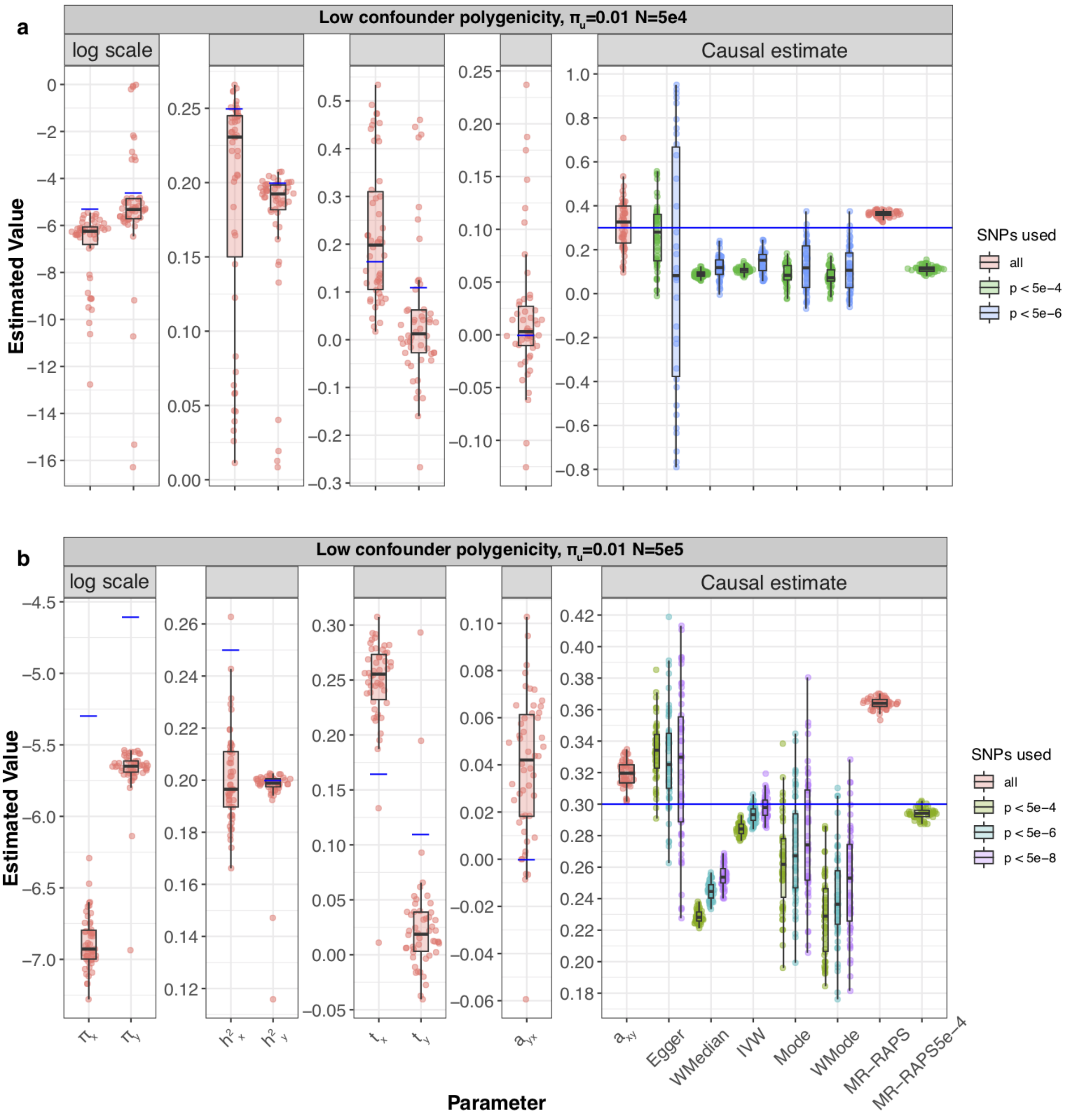

Supplementary Figure 10: Simulation results where the polygenicity of the confounder is reduced. Modified Sina-boxplots representing the distribution of parameter estimates from 50 different data generations. For each generation, standard MR methods as well as our LHC-MR were used to estimate a causal effect. In the boxplots, the lower and upper hinges correspond to the first and third quartiles, the middle bar corresponds to the median, whereas the upper whisker is the largest dataset estimate smaller than 1.5∗inter-quartile range above the third quartile. The lower whisker is defined analogously. The true values of the parameters used in the data generations are represented by the blue dots/lines. In this figure, the polygenicity for U is decreased in the form of lower $\pi_u = 0.01$. **a** Results for smaller sample size of $n_x = n_y = 50,000$. **b** Results for larger sample size of $n_x = n_y = 500,000$.

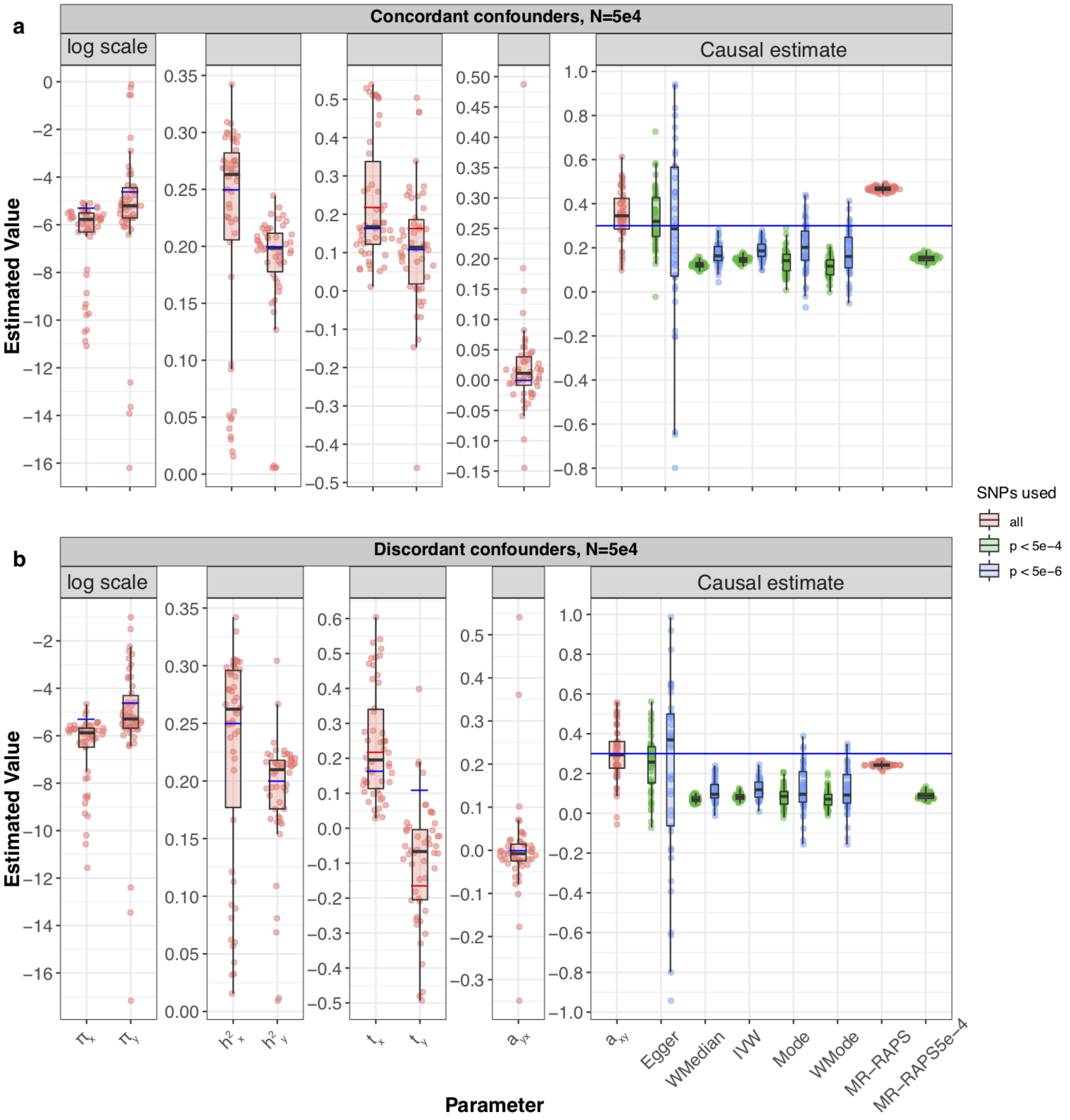

Supplementary Figure 11: Simulation results where there are two underlying confounders, once with concordant and another with discordant effects on the exposure-outcome pair. Modified Sina-boxplots representing the distribution of parameter estimates from 50 different data generations. For each generation, standard MR methods as well as our LHC-MR were used to estimate a causal effect. In the boxplots, the lower and upper hinges correspond to the first and third quartiles, the middle bar corresponds to the median, whereas the upper whisker is the largest dataset estimate smaller than 1.5∗inter-quartile range above the third quartile. The lower whisker is defined analogously. The true values of the parameters used in the data generations are represented by the blue dots/lines. **a** The underlying data generations have two concordant heritable confounders U_1 and U_2 with positive effects on traits X and Y. **b** The data generations have two discordant heritable confounders with $t_x^{(1)} = 0.16, t_y^{(1)} = 0.11$ shown as blue dots and $t_x^{(2)} = 0.22, t_y^{(2)} = -0.16$ shown as red dots.

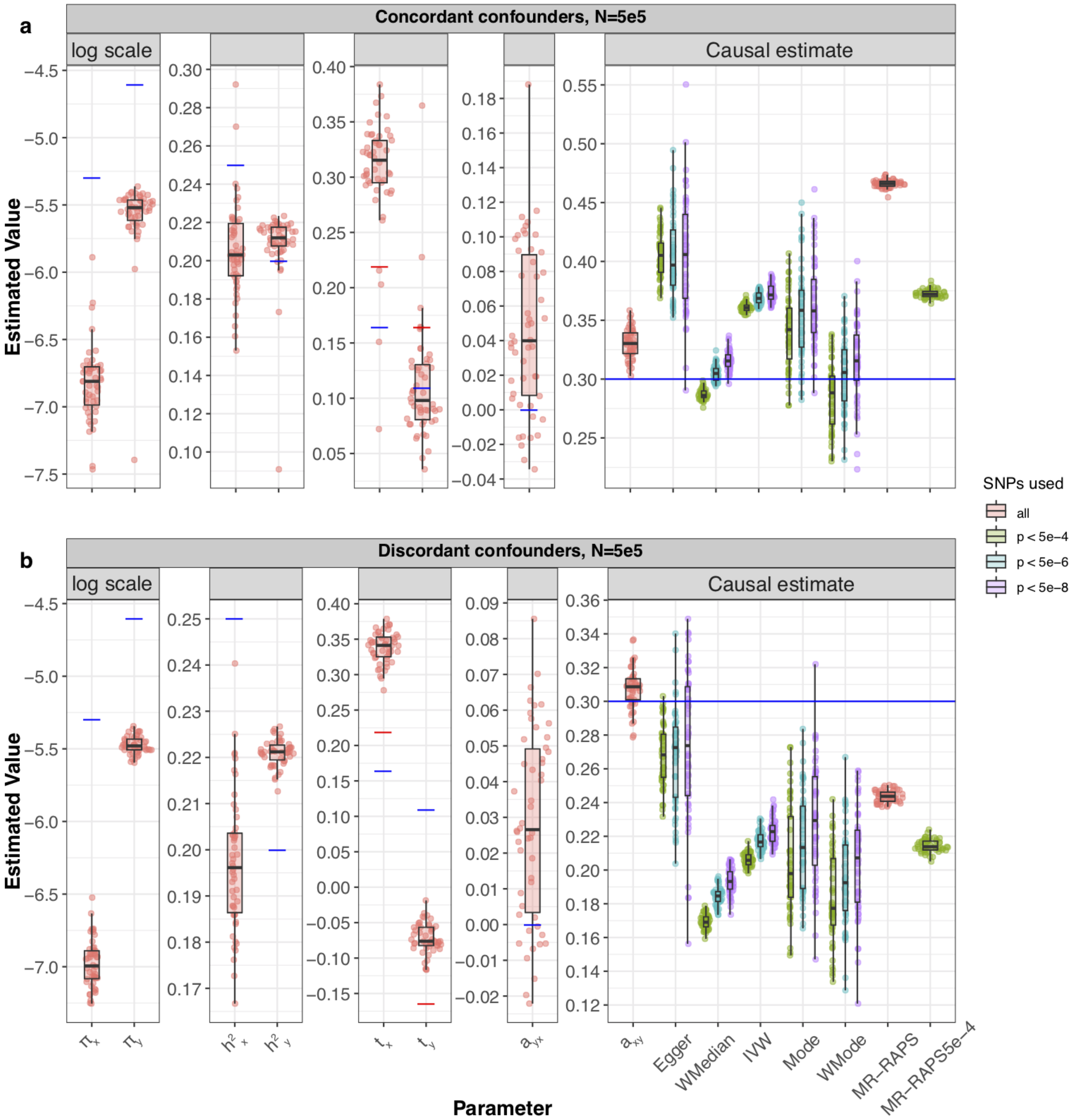

Supplementary Figure 12: Simulation results where there are two underlying confounders, once with concordant and another with discordant effects on the exposure-outcome pair. Modified Sina-boxplots representing the distribution of parameter estimates from 50 different data generations. For each generation, standard MR methods as well as our LHC-MR were used to estimate a causal effect. In the boxplots, the lower and upper hinges correspond to the first and third quartiles, the middle bar corresponds to the median, whereas the upper whisker is the largest dataset estimate smaller than 1.5∗inter-quartile range above the third quartile. The lower whisker is defined analogously. The true values of the parameters used in the data generations are represented by the blue dots/lines. **a** The underlying data generations have two concordant heritable confounders U_1 and U_2 with positive effects on traits X and Y. **b** The data generations have two discordant heritable confounders with $t_x^{(1)} = 0.16, t_y^{(1)} = 0.11$ shown as blue dots and $t_x^{(2)} = 0.22, t_y^{(2)} = -0.16$ shown as red dots.

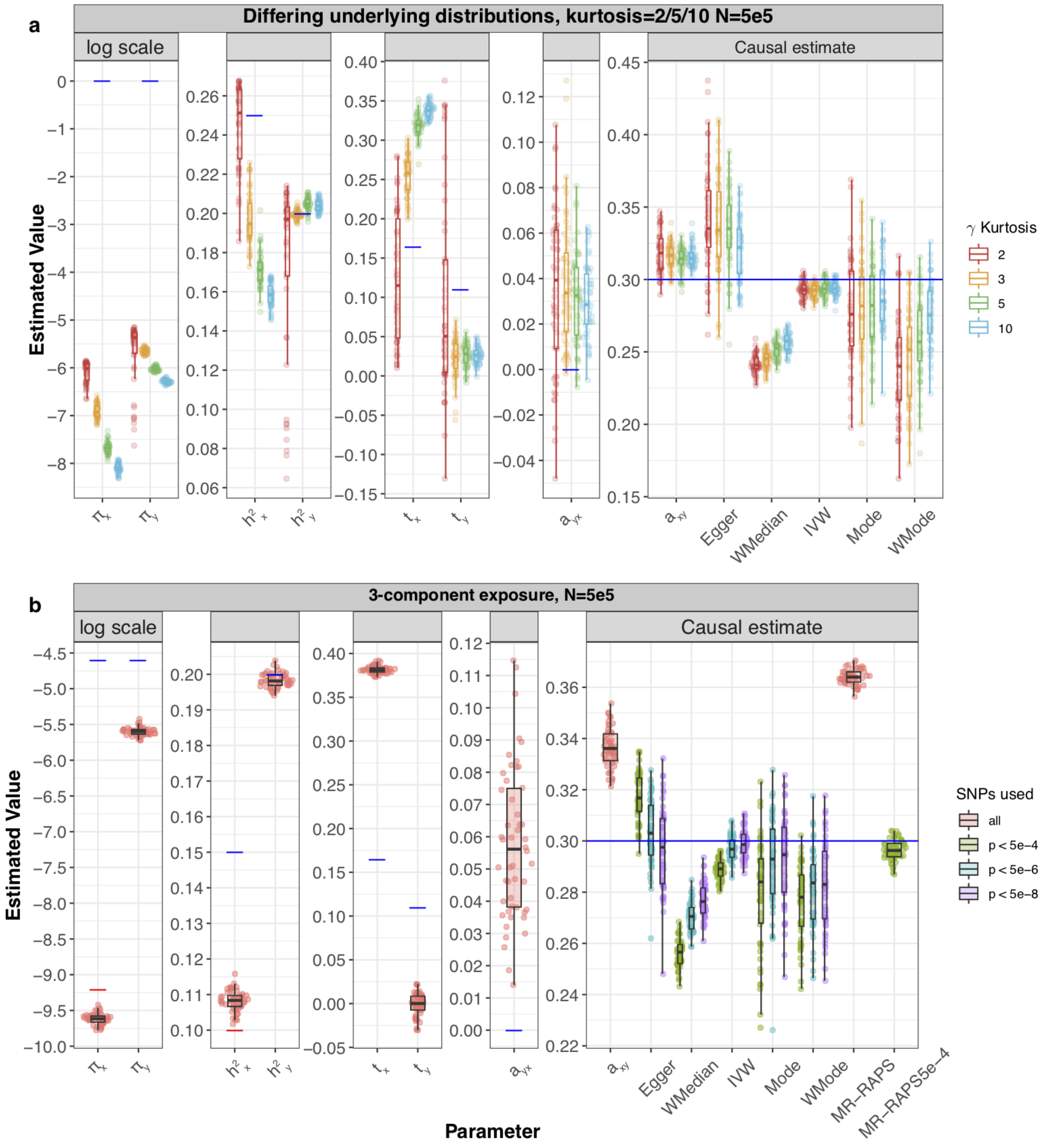

Supplementary Figure 13: Simulation results under various scenarios. These modified Sina-boxplots represent the distribution of parameter estimates from 50 different data generations under various conditions. For each generation, standard MR methods as well as our LHC-MR were used to estimate a causal effect. In the boxplots, the lower and upper hinges correspond to the first and third quartiles, the middle bar corresponds to the median, whereas the upper whisker is the largest dataset estimate smaller than 1.5∗inter-quartile range above the third quartile. The lower whisker is defined analogously. The true values of the parameters used in the data generations are represented by the blue dots/lines. **a** The different coloured boxplots represent the underlying non-normal distribution used in the simulation of the three $\gamma_x, \gamma_x, \gamma_u$ vectors associated to their respective traits. The Pearson distributions had the same 0 mean and skewness, however their kurtosis ranged between 2 and 10, including the kurtosis of 3, which corresponds to a normal distribution assumed by our model. The standard MR results reported had IVs selected with a p-value threshold of 5×10^{-6}. **b** Addition of a third component for exposure X, while decreasing the strength of U. True parameter values are in colour, blue and red for each component ($\pi_{x1} = 1 \times 10^{-4}, \pi_{x2} = 1 \times 10^{-2}, h^2_{x1} = 0.15, h^2_{x2} = 0.1$).

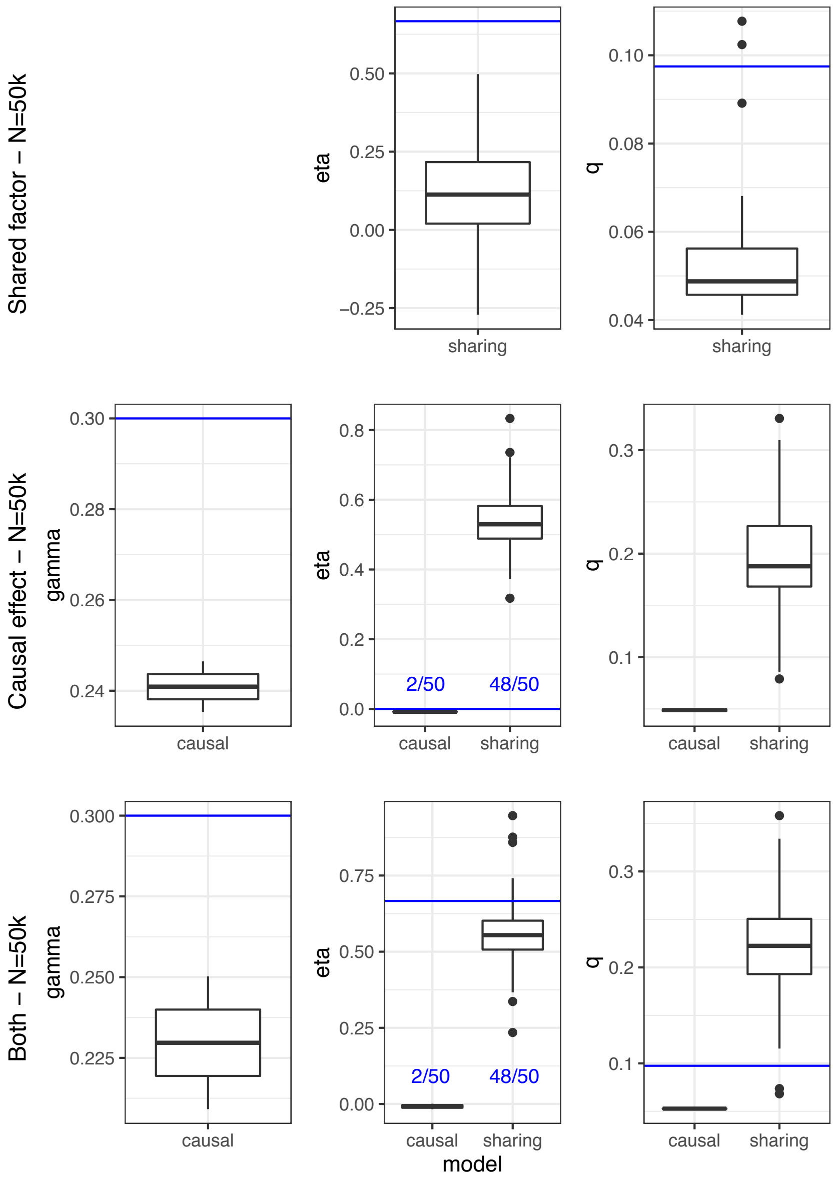

Supplementary Figure 14: Running CAUSE on LHC-MR simulated data under the standard settings. Boxplots of the parameter estimation of CAUSE on LHC-simulated data ($n_x = n_y = 50,000$), with 50 different data generations under three different scenarios: presence of a shared factor only, presence of a causal effect only, presence of both. In the boxplots, the lower and upper hinges correspond to the first and third quartiles, the middle bar corresponds to the median, whereas the upper whisker is the largest dataset estimate smaller than 1.5∗inter-quartile range above the third quartile. The lower whisker is defined analogously. CAUSE returns two possible models with a respective p-value, the sharing and the causal model, where the causal mode is the significant of the two. When only an underlying shared factor was present in the simulated data, CAUSE had no significant causal estimates. With a true underlying causal effect, or when both an underlying causal effect and a shared factor was present, the causal model was significant only 4% of the simulations.

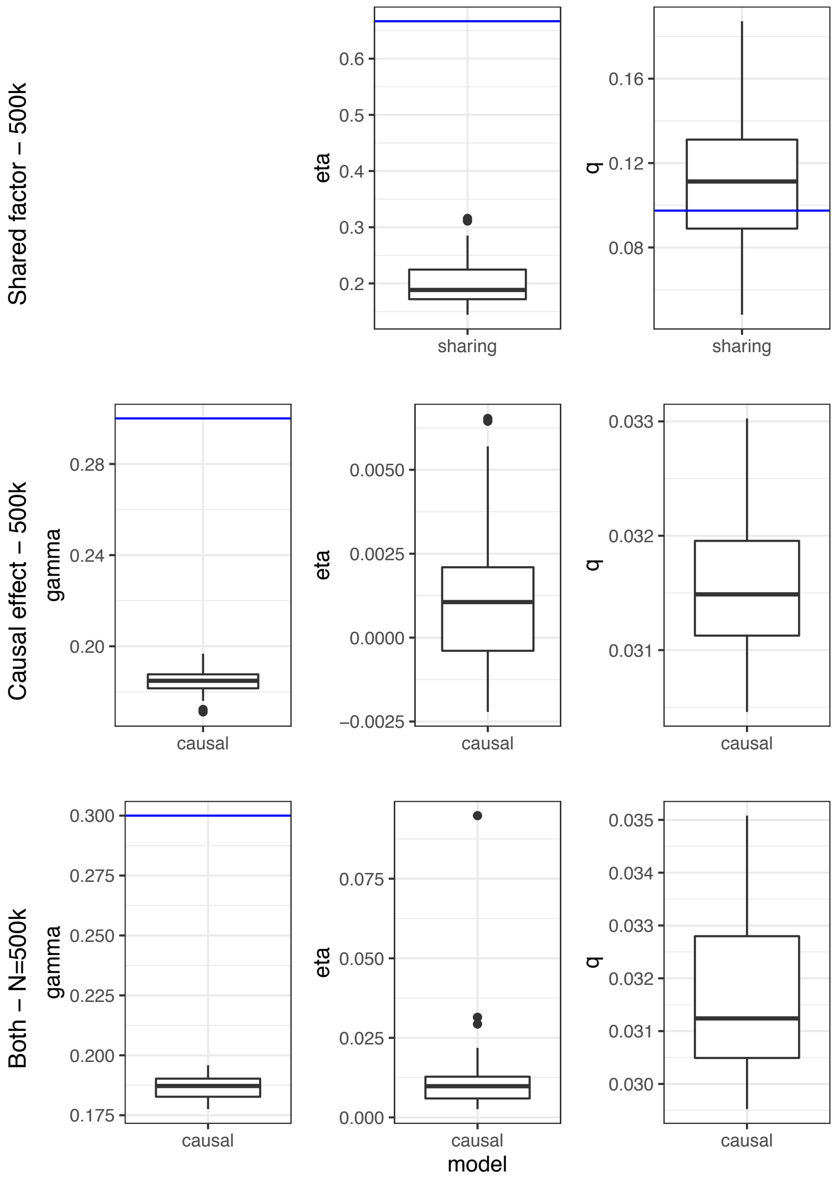

Supplementary Figure 15: Running CAUSE on LHC-MR simulated data under the standard settings. Boxplots of the parameter estimation of CAUSE on LHC-simulated data ($n_x = n_y = 500,000$), with 50 different data generations under three different scenarios: presence of a shared factor only, presence of a causal effect only, presence of both. In the boxplots, the lower and upper hinges correspond to the first and third quartiles, the middle bar corresponds to the median, whereas the upper whisker is the largest dataset estimate smaller than 1.5∗inter-quartile range above the third quartile. The lower whisker is defined analogously. CAUSE returns two possible models with a respective p-value, the sharing and the causal model, where the causal mode is the significant of the two. When only an underlying shared factor was present in the simulated data, CAUSE had no significant causal estimates. With a true underlying causal effect, or when both an underlying causal effect and a shared factor was present, the causal model was significant 100% of the simulations.

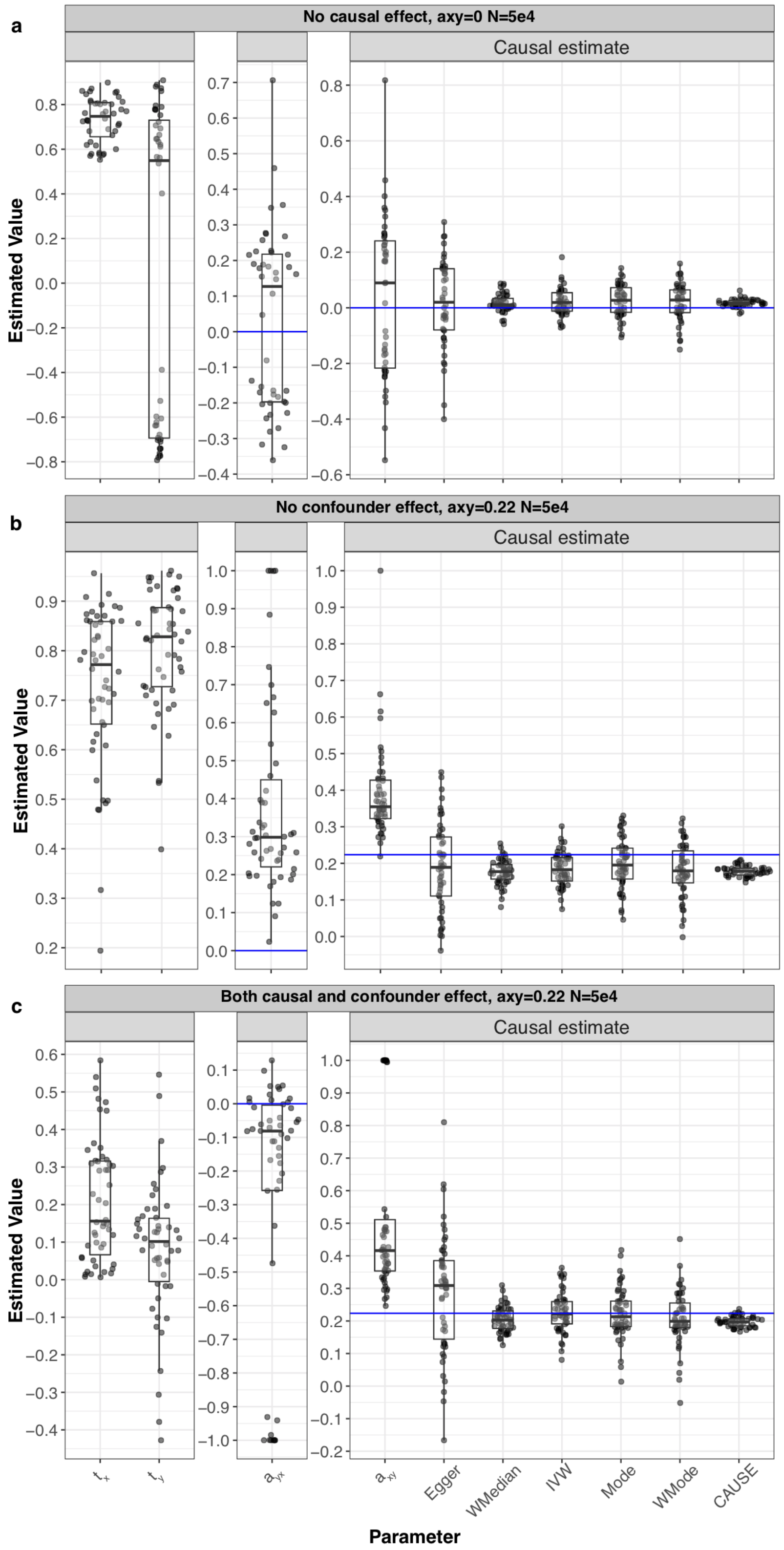

Supplementary Figure 16: Running LHC-MR on CAUSE simulated data under various scenarios. Modified Sina-boxplots representing the distribution of parameter estimates from LHC-MR of 50 different data generations using the CAUSE framework. For each generation, standard MR methods, CAUSE as well as our LHC-MR were used to estimate a causal effect. In the boxplots, the lower and upper hinges correspond to the first and third quartiles, the middle bar corresponds to the median, whereas the upper whisker is the largest dataset estimate smaller than 1.5∗inter-quartile range above the third quartile. The lower whisker is defined analogously. The true values of the parameters used in the data generations are represented by the blue dots/lines. **a** CAUSE data was generated with no causal effect but with a shared factor with an η value of ~ 0.22. CAUSE chooses a sharing model 100% of the time with no estimate for a causal effect. **b** CAUSE is simulated with causal effect but with no shared factor. **c** CAUSE is simulated with both a causal effect and a shared factor.

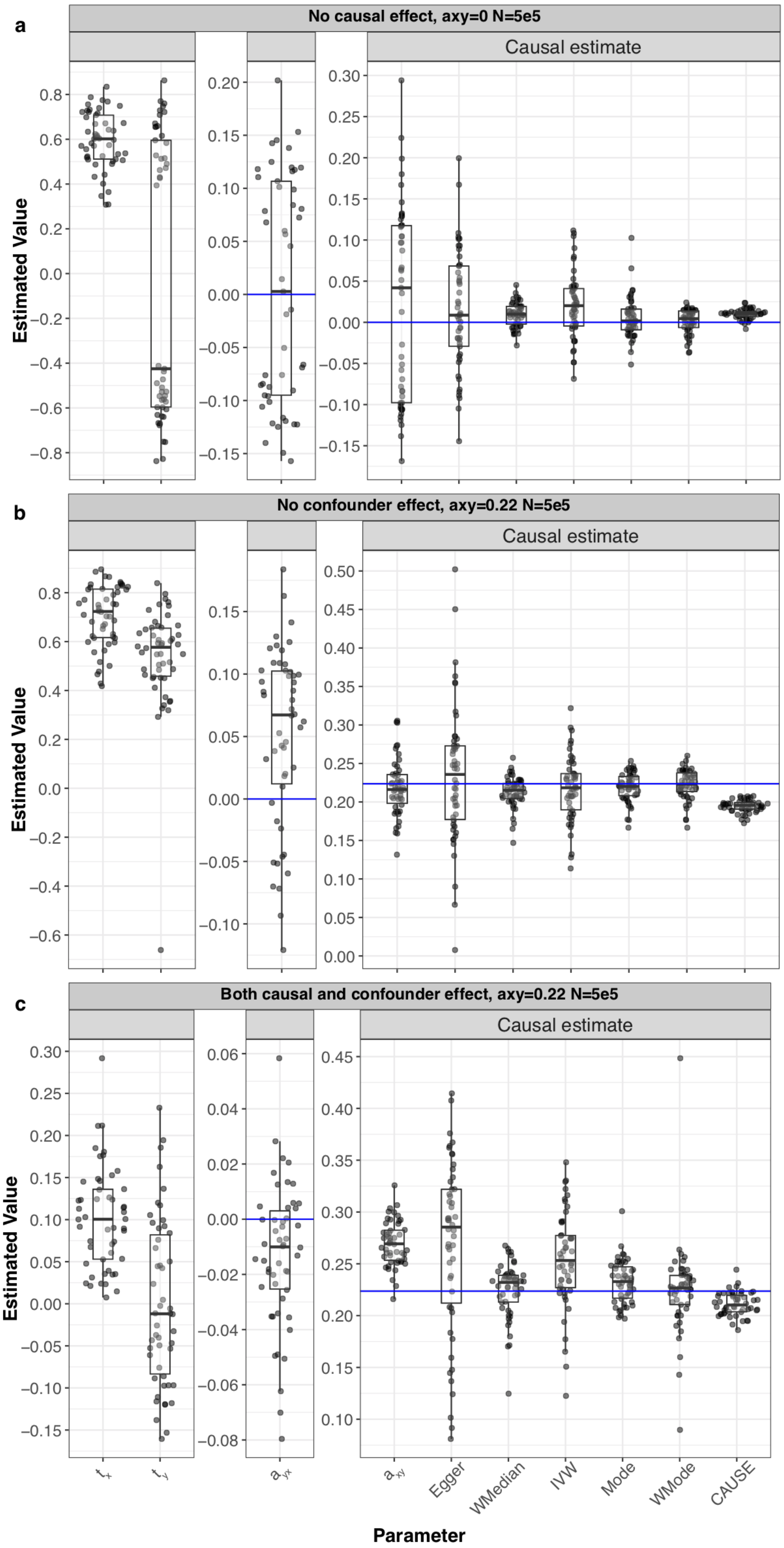

Supplementary Figure 17: Running LHC-MR on CAUSE simulated data under various scenarios. Modified Sina-boxplots representing the distribution of parameter estimates from LHC-MR of 50 different data generations using the CAUSE framework. For each generation, standard MR methods, CAUSE as well as our LHC-MR were used to estimate a causal effect. In the boxplots, the lower and upper hinges correspond to the first and third quartiles, the middle bar corresponds to the median, whereas the upper whisker is the largest dataset estimate smaller than 1.5∗inter-quartile range above the third quartile. The lower whisker is defined analogously. The true values of the parameters used in the data generations are represented by the blue dots/lines. **a** CAUSE data was generated with no causal effect but with a shared factor with an η value of ~ 0.22. **b** CAUSE is simulated with causal effect but with no shared factor. **c** CAUSE is simulated with both a causal effect and a shared factor. LHC-MR seems to exhibit a bimodal effect at first glance, but the two peaks are not connected.

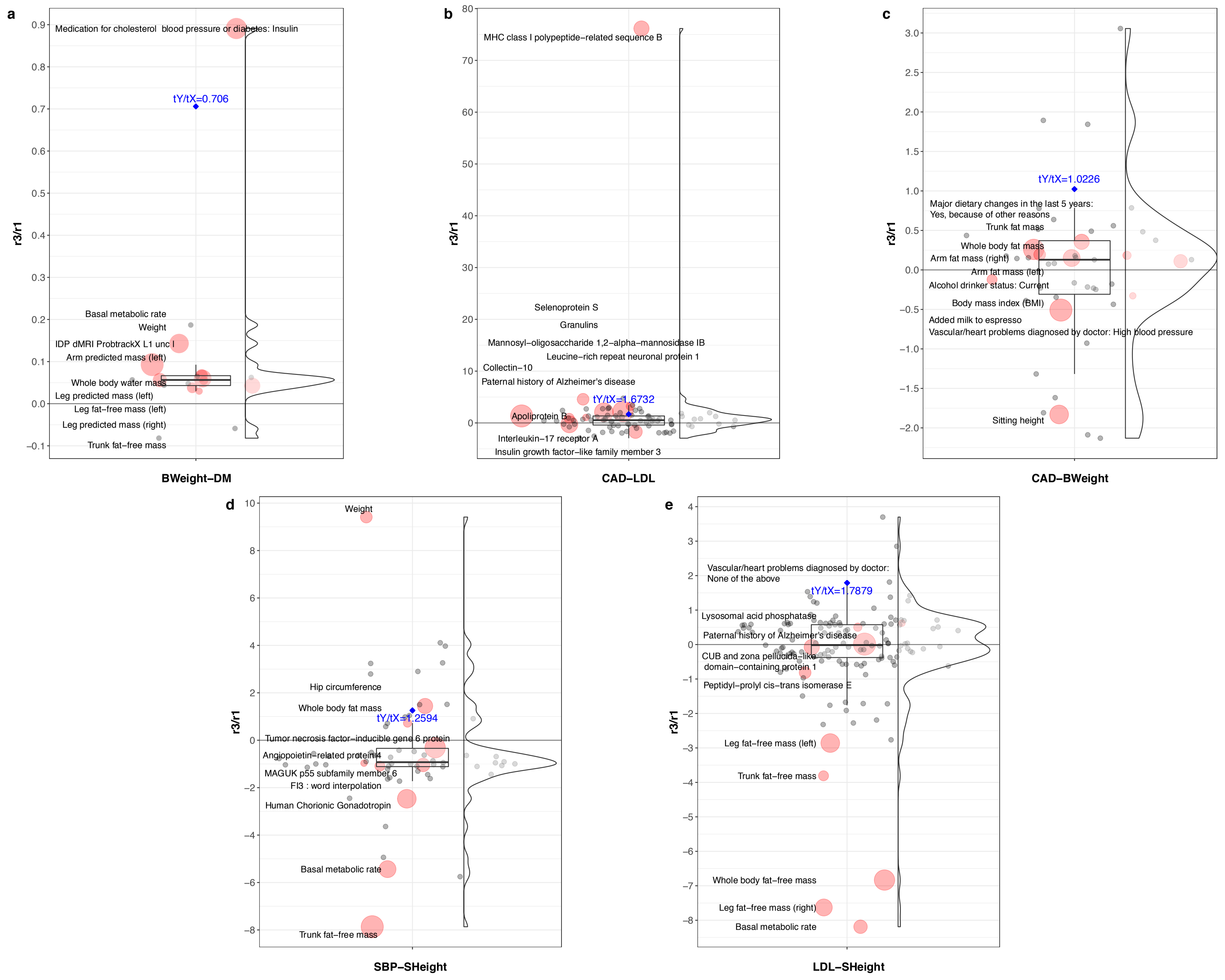
a
b
c
d
e
r3/r1
BWeight–DM
tY/tX=0.706
Medication for cholesterol blood pressure or diabetes: Insulin
Basal metabolic rate
Weight
IDP dMRI ProbtrackX L1 unc l
Arm predicted mass (left)
Whole body water mass
Leg predicted mass (left)
Leg fat–free mass (left)
Leg predicted mass (right)
Trunk fat–free mass
CAD–LDL
tY/tX=1.6732
MHC class I polypeptide–related sequence B
Selenoprotein S
Granulins
Mannosyl–oligosaccharide 1,2–alpha–mannosidase IB
Leucine–rich repeat neuronal protein 1
Collectin–10
Paternal history of Alzheimer's disease
Apolipoprotein B
Interleukin–17 receptor A
Insulin growth factor–like family member 3
CAD–BWeight
tY/tX=1.0226
Major dietary changes in the last 5 years:
Yes, because of other reasons
Trunk fat mass
Whole body fat mass
Arm fat mass (right)
Arm fat mass (left)
Alcohol drinker status: Current
Body mass index (BMI)
Added milk to espresso
Vascular/heart problems diagnosed by doctor: High blood pressure
Sitting height
SBP–SHeight
tY/tX=1.2594
Weight
Hip circumference
Whole body fat mass
Tumor necrosis factor–inducible gene 6 protein
Angiopoietin–related protein 4
MAGUK p55 subfamily member 6
FI3 : word interpolation
Human Chorionic Gonadotropin
Basal metabolic rate
Trunk fat–free mass
LDL–SHeight
tY/tX=1.7879
Vascular/heart problems diagnosed by doctor:
None of the above
Lysosomal acid phosphatase
Paternal history of Alzheimer's disease
CUB and zona pellucida–like
domain–containing protein 1
Peptidyl–prolyl cis–trans isomerase E
Leg fat–free mass (left)
Trunk fat–free mass
Whole body fat–free mass
Leg fat–free mass (right)
Basal metabolic rate

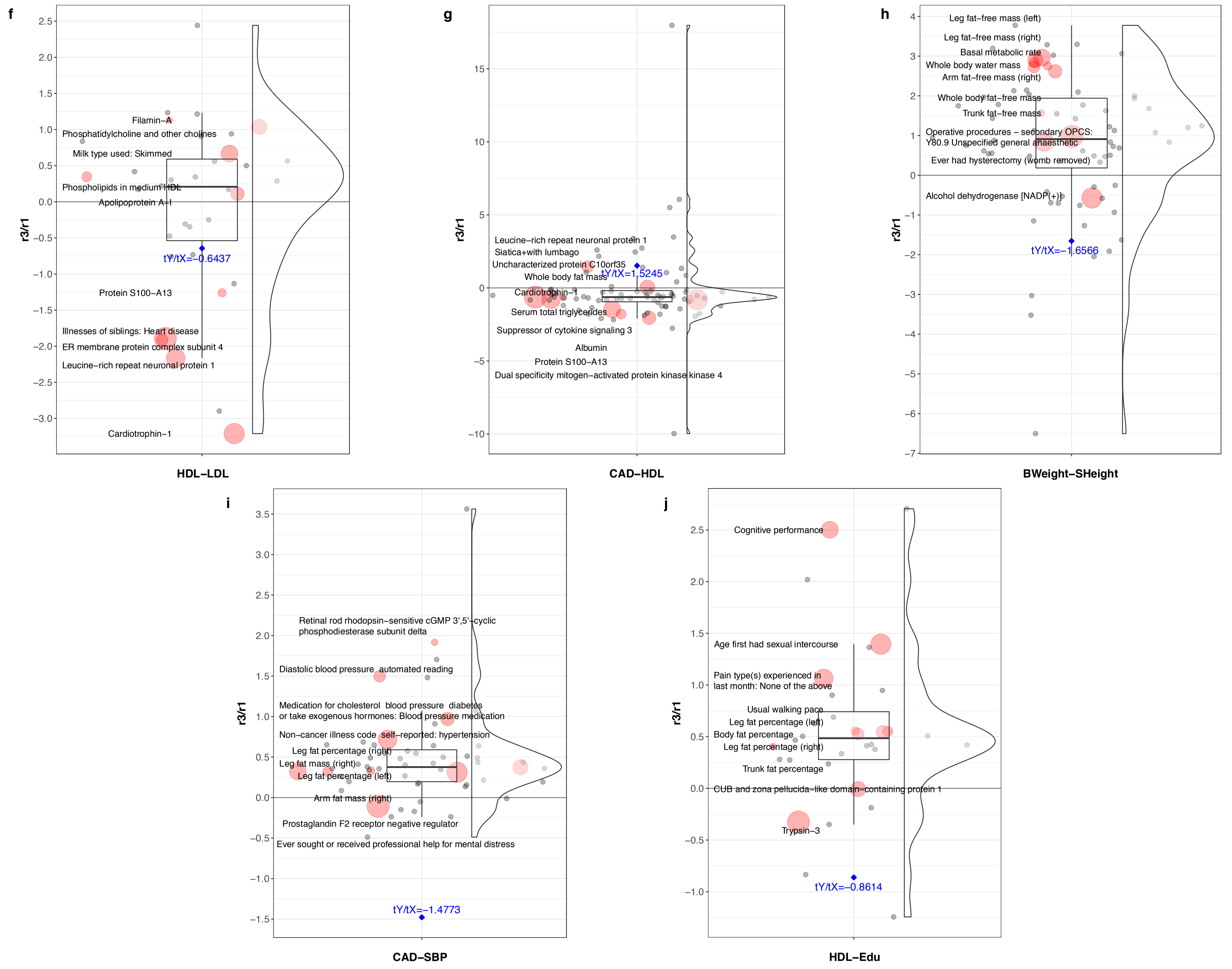

Supplementary Figure 18: Confounder effects obtained from EpiGraphDB plotted as a modified Sina-boxplot with the r_3/r_1 ratio on the y-axis. The blue diamonds represent the t_y/t_x ratio derived from the LHC model for that trait pair, also reported in blue text. Labelled dots in red and their varying size show the ten largest confounder traits in terms of their absolute effect product on the two traits, whereas grey dots represent the rest of the confounder traits found by EpigraphDB. In the boxplots, the lower and upper hinges correspond to the first and third quartiles, the middle bar corresponds to the median, whereas the upper whisker is the largest dataset point smaller than 1.5∗inter-quartile range above the third quartile. The lower whisker is defined analogously. Confounder effects of trait pairs: **a** Birth Weight - Diabetes Mellitus, **b** Coronary Artery Disease - Low-density Lipoprotein, **c** Coronary Artery Disease - Birth Weight, **d** Systolic Blood Pressure - Standing Height, **e** Low-density Lipoprotein - Standing Height, **f** High-density Lipoprotein - Low-density Lipoprotein, **g** Coronary Artery Disease - High-density Lipoprotein, **h** Birth Weight - Standing Height, **i** Coronary Artery Disease - Systolic Blood Pressure, **j** High-density Lipoprotein - Years of Education

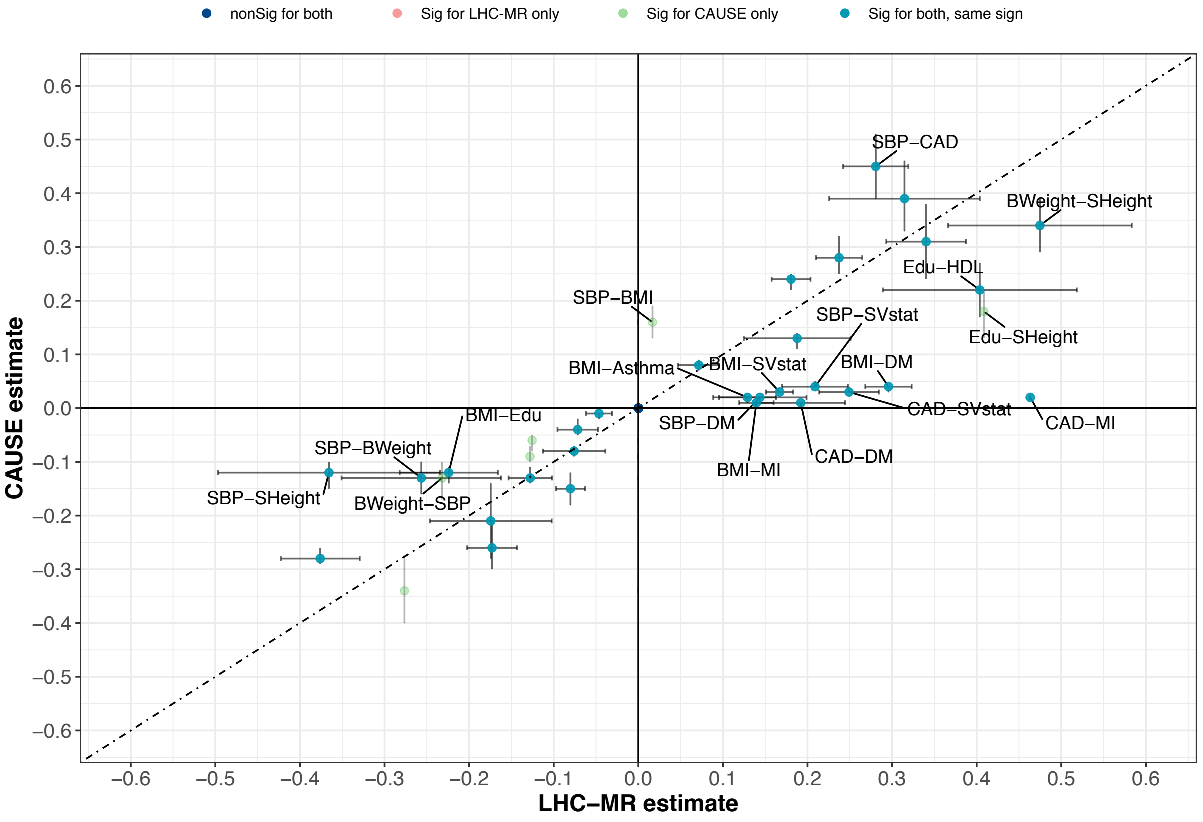

Supplementary Figure 19: A scatter plot of the causal effect estimates between LHC-MR and CAUSE. To improve visibility, non-significant estimates by both methods are placed at the origin, while significant estimates by both methods appear on the diagonal with 95% CI error bars for LHC-MR causal estimates, and 95% credible interval error bars for CAUSE estimates. Labelled pairs are those with an estimate difference greater than 0.1.

Supplementary Tables

UKBB ID / Data Origin	Trait Name	Abbreviation	Sample Size	PMID
**845**	Age completed full time education	Edu	240,547	25826379
**21001_irnt**	Body mass index (BMI)	BMI	359,983	25826379
**2443**	Diabetes diagnosed by doctor	DM	360,192	25826379
**20002_1075**	Non-cancer illness code, self-reported: heart attack/myocardial infarction	MI	361,141	25826379
**20002_1111**	Non-cancer illness code, self-reported: asthma	Asthma	361,141	25826379
**2887**	Number of cigarettes previously smoked daily	PSmoke	84,456	25826379
**20022_irnt**	Birth weight	BWeight	205,475	25826379
**50_irnt**	Standing height	SHeight	360,388	25826379
**4080**	Systolic blood pressure, automated reading	SBP	340,159	25826379
**20003_1140861958**	Treatment/medication code: simvastatin	SVstat	361,141	25826379
**30780_irnt**	LDL Cholesterol	LDL	343,621	25826379
**30760_irnt**	HDL Cholesterol	HDL	315,133	25826379
**UKBB + CARDIoGRAMplusC4D**	Coronary Artery Disease	CAD	380,831	29212778

Supplementary Table 1: Details of the origin study of each trait, its abbreviation used in this paper, the sample size of the study for that trait, as well as the PubMed article ID.

a

LHC-MR vs MR-Egger

	Sig+	Sig–	nonSig
**Sig+**	10	0	27
**Sig–**	0	2	35
**nonSig**	0	2	56

b

LHC-MR vs WMedian

	Sig+	Sig–	nonSig
**Sig+**	21	1	15
**Sig–**	0	12	25
**nonSig**	1	6	51

c

LHC-MR vs IVW

	Sig+	Sig–	nonSig
**Sig+**	23	0	14
**Sig–**	0	17	20
**nonSig**	2	5	51

d

LHC-MR vs Mode

	Sig+	Sig–	nonSig
**Sig+**	12	0	25
**Sig–**	0	2	35
**nonSig**	0	0	58

e

LHC-MR vs WMode

	Sig+	Sig–	nonSig
**Sig+**	13	3	21
**Sig–**	0	7	30
**nonSig**	0	2	56

f

MR-Egger vs WMode

	Sig+	Sig–	nonSig
**Sig+**	10	0	0
**Sig–**	0	1	3
**nonSig**	3	11	104

Supplementary Table 2: **Cross tables between LHC-MR and various standard MR methods comparing the significance and sign of each respective causal estimate**. **f** shows a cross table between the two-least correlated MR methods in terms of their estimates.

Pair	$\alpha_{x \to y}$	p-value	γ	IVW $\alpha_{x \to y}$	p-value
BMI-Asthma	0.1290	4.99E-14	0.02 (0.01, 0.02)	0.0593	1.00E-08
BMI-DM	0.2958	1.07E-99	0.04 (0.03, 0.04)	0.2447	2.25E-140
BMI-SBP	0.1878	5.55E-09	0.13 (0.11, 0.14)	0.1547	1.11E-24
BMI-SVstat	0.1670	2.08E-91	0.03 (0.03, 0.03)	0.1570	4.26E-63
BMI-MI	0.1396	1.67E-41	0.01 (0.01, 0.01)	0.1027	9.16E-32
BWeight-SHeight	0.4748	9.60E-18	0.34 (0.29, 0.39)	0.2959	8.01E-10
SHeight-BWeight	0.1806	1.93E-53	0.24 (0.22, 0.25)	0.1803	7.21E-86
SBP-DM	0.1437	3.17E-07	0.02 (0.01, 0.02)	0.0697	3.69E-07
DM-SVstat	0.3147	4.11E-12	0.39 (0.33, 0.46)	0.2524	1.28E-16
SHeight-Edu	0.0715	8.42E-09	0.08 (0.07, 0.09)	0.0643	2.28E-21
SBP-SVstat	0.2089	4.84E-26	0.04 (0.04, 0.05)	0.1853	1.46E-52
Edu-HDL	0.4037	5.25E-12	0.22 (0.17, 0.27)	0.2848	4.06E-08
BMI-CAD	0.2373	2.37E-64	0.28 (0.25, 0.32)	0.1800	2.42E-53
CAD-DM	0.1920	5.92E-13	0.01 (0.01, 0.01)	0.0659	0.002455431
DM-CAD	0.4283	5.60E-19	1.95 (1.26, 2.64)	0.1796	4.15E-05
SBP-CAD	0.2807	2.86E-46	0.45 (0.39, 0.51)	0.2500	9.77E-24
CAD-SVstat	0.2491	8.82E-44	0.03 (0.03, 0.04)	0.3077	1.15E-25
CAD-MI	0.4634	0	0.02 (0.02, 0.02)	0.4191	3.07E-285
LDL-CAD	0.3402	1.17E-45	0.31 (0.24, 0.38)	0.2014	8.56E-27
BMI-Edu	-0.2241	3.74E-14	-0.12 (-0.14, -0.11)	-0.1892	6.15E-35
SHeight-BMI	-0.1278	1.40E-22	-0.13 (-0.14, -0.11)	-0.0854	9.01E-23
SBP-BWeight	-0.2565	9.85E-08	-0.13 (-0.16, -0.1)	-0.1646	1.20E-11
SBP-SHeight	-0.3657	4.81E-08	-0.12 (-0.15, -0.1)	-0.0967	0.004422636
SHeight-SBP	-0.0759	5.74E-05	-0.08 (-0.09, -0.07)	-0.0652	1.25E-15
SHeight-SVstat	-0.0465	4.76E-09	-0.01 (-0.02, -0.01)	-0.0328	6.78E-12
BMI-HDL	-0.3760	3.54E-56	-0.28 (-0.29, -0.26)	-0.3630	3.17E-111
SHeight-LDL	-0.0716	4.26E-09	-0.04 (-0.05, -0.02)	-0.0298	5.07E-06
BWeight-CAD	-0.1745	2.05E-06	-0.21 (-0.28, -0.14)	-0.0978	2.83E-05
SHeight-CAD	-0.0802	3.72E-20	-0.15 (-0.18, -0.12)	-0.0482	2.18E-12
HDL-CAD	-0.1729	7.00E-31	-0.26 (-0.3, -0.21)	-0.0778	5.45E-10

Supplementary Table 3: Table comparing the causal estimates of LHC-MR, CAUSE, and IVW for trait pairs that had a significant causal effect in LHC-MR and CAUSE. The column showing the gamma (causal effect) estimate of the CAUSE method also reports its 95% credible intervals. A complete table for all the studied pairs is found in the Supplementary Data 4.
