# Supplementary material for: Simultaneous estimation of bi-directional causal effects and heritable confounding from GWAS summary statistics
Source: Nat Commun. 2021 Dec 14;12:7274. doi: 10.1038/s41467-021-26970-w (PMC8671515; doi:10.1038/s41467-021-26970-w)
Supplement: Supplementary file 3 — Description of supplementary files [file 41467_2021_26970_MOESM3_ESM.pdf]

Description of Additional Supplementary Files

Supplementary Data 1 - Simulation summary: The bias, variance, and RMSE of each estimated parameter is reported for the various scenarios. The same values are reported for standard MR methods with their various thresholding limits.

Supplementary Data 2 - LHC-MR Real data main results: LHC-MR comprehensive results of the main parameter estimations for each trait pair.

Supplementary Data 3 - LHC-MR Real data alternate results: LHC-MR comprehensive results of the alternate parameter estimations for each trait pair.

Supplementary Data 4 - All methods - causal estimates: Causal estimates of LHC-MR, standard MR methods, and CAUSE (gamma, eta and q are reported with their 95% credible intervals) for each trait pair (in both directions) with p-value and SE reported.

Supplementary Data 5 - Causal estimate comparison: Causal estimates of LHC-MR, standard MR methods, and CAUSE for trait pairs with sufficient heritability ($> 2.5\%$) (in both directions) with p-value and SE reported. Significance comparison between LHC-MR and standard MR methods is reported (t-test).

Supplementary Data 6 - LDSC comparison: LDsc results for each trait pair, heritability of each trait and genetic correlation are reported. Genetic correlation calculations based on LHC-MR estimates are also reported.

**Parameter**	**Explanation**
Pair	trait pair
EXP	trait used as exposure (X)
OUT	trait used as outcome (Y)
piX	polygenicity: proportion of non-zero multivariable causal effects among all sequence variants directly influencing exposure X
piY	polygenicity: proportion of non-zero multivariable causal effects among all sequence variants directly influencing outcome Y
h2X	direct heritability on exposure X
h2Y	direct heritability on outcome Y
tX	confounder effect on X
tY	confounder effect on Y
axy	causal effect from X to Y
ayx	causal effect from Y to X
iXY	parameter equivalent to the LDSC cross-trait intercept
[parameter]_JKse	standard error of each parameter from the 200 block jackknife
[parameter]_JKpval	calculated p-value of each parameter from the 200 block jackknife
tot_h2X	total heritability of the exposure (direct + indirect), calculated as: h2X + (tX + (ayx*tY))$\hat{2}$ + h2Y*ayx$\hat{2}$
tot_h2Y	total heritability of the outcome (direct + indirect), calculated as: h2Y + (tY + (axy*tX))$\hat{2}$ + h2X*axy$\hat{2}$

low_h2X<br>low_h2Y<br>SingleTrait_[parameter]	whether the total heritability of the exposure is less than 2.5%<br>whether the total heritability of the outcome is less than 2.5%<br>parameter estimates from running the single trait analysis (no confounder, no causal relationships) : total heritability (h2X and h2Y), polygenicity (piX and piY) and LDSC intercept (iX and iY) for each trait
[MRmethod]E4<br><br>X-compatible<br>X-plausible<br>Y-compatible<br>Y-plausible	Bias/Variance/RMSE calculated for the estimates when the MR method used IVs with a p-value threshold of 5e-4<br><br>boolean indicating if the parameters calculated from the switching of X and U fall within our parameter ranges<br>boolean indicating if the parameters calculated from the switching of X and U is plausible (direct heritability larger than indirect heritability)<br>boolean indicating if the parameters calculated from the switching of Y and U fall within our parameter ranges<br>boolean indicating if the parameters calculated from the switching of X and U is plausible (direct heritability larger than indirect heritability)
tY:tX<br>sig_axy<br>sig_U<br>CAUSE_winning model<br>gamma<br>eta<br>q	ratio of tY to tX (in red when confounder's effect and causal effect are in opposite directions)<br>boolean indicating if the causal effect estimate is significant<br>boolean indicating if both tX and tY are significant (green when TRUE)<br>either "Causal" or "Sharing"<br>CAUSE causal effect estimate and 95% credible interval (only if the winning model is "Causal")<br>CAUSE estimate for the effect of correlated pleiotropy (equivalent to LHC-MR tY/tX ratio) and 95% credible interval<br>CAUSE estimate for the proportion of variants exhibiting correlated pleiotropy and 95% credible interval
sum_[Method]<br><br><br><br><br>Qstat_pvalue<br>tstat_[MRmethod]<br>tstat_[MRmethod]_pval<br>same_est	Indicating whether the estimate of the method is insignificant 0, significant positive + or significant negative -. Comparing the methods for each trait pair, green indicates + estimates, orange indicates - estimates, grey for 0 estimates, and red for the estimates with an opposite sign to LHC-MR<br>P-value of the Q-test done to compare how alike the MR estimates are. Values less than 0.05/156 are discordant<br>T-stat value for comparing the LHC-MR estimate with that of MR methods<br>P-value of the t-test done to compare the HC-MR estimate with that of MR methods. Values greater than 0.05/156 are similar<br>Counts how many times LHC-MR had similar (same) causal effect estimates with the 5 MR methods
gcorr	genetic correlation, calculated from LDsc

gcorr_se	SE of the genetic correlation
lower_gcorr_LHC	lower bound of the 200 genetic correlation estimates obtained from the parameter estimates of the 200 block jackknife
upper_gcorr_LHC	upper bound of the 200 genetic correlation estimates obtained from the parameter estimates of the 200 block jackknife
mean_gcorr_LHC	mean of the 200 genetic correlation estimates obtained from the parameter estimates of the 200 block jackknife
sd_gcorr_LHC	SD 200 genetic correlation estimates obtained from the parameter estimates of the 200 block jackknife
