# Supplementary material for: Simultaneous estimation of bi-directional causal effects and heritable confounding from GWAS summary statistics
Source: Nat Commun. 2021 Dec 14;12:7274. doi: 10.1038/s41467-021-26970-w (PMC8671515; doi:10.1038/s41467-021-26970-w)
Supplement: Supplementary file 10 — Reporting Summary [file 41467_2021_26970_MOESM10_ESM.pdf]

nature portfolio

Corresponding author(s): Zoltán Kutalik

Last updated by author(s): Sep 29, 2021

Reporting Summary

Nature Portfolio wishes to improve the reproducibility of the work that we publish. This form provides structure for consistency and transparency in reporting. For further information on Nature Portfolio policies, see our Editorial Policies and the Editorial Policy Checklist.

Statistics

For all statistical analyses, confirm that the following items are present in the figure legend, table legend, main text, or Methods section.

n/a	Confirmed	
☐	☒	The exact sample size (n) for each experimental group/condition, given as a discrete number and unit of measurement
☐	☒	A statement on whether measurements were taken from distinct samples or whether the same sample was measured repeatedly
☐	☒	The statistical test(s) used AND whether they are one- or two-sided<br>*Only common tests should be described solely by name; describe more complex techniques in the Methods section.*
☐	☒	A description of all covariates tested
☐	☒	A description of any assumptions or corrections, such as tests of normality and adjustment for multiple comparisons
☐	☒	A full description of the statistical parameters including central tendency (e.g. means) or other basic estimates (e.g. regression coefficient) AND variation (e.g. standard deviation) or associated estimates of uncertainty (e.g. confidence intervals)
☐	☒	For null hypothesis testing, the test statistic (e.g. F, t, r) with confidence intervals, effect sizes, degrees of freedom and P value noted<br>*Give P values as exact values whenever suitable.*
☒	☐	For Bayesian analysis, information on the choice of priors and Markov chain Monte Carlo settings
☐	☒	For hierarchical and complex designs, identification of the appropriate level for tests and full reporting of outcomes
☐	☒	Estimates of effect sizes (e.g. Cohen's d, Pearson's r), indicating how they were calculated

Our web collection on statistics for biologists contains articles on many of the points above.

Software and code

Policy information about availability of computer code

Data collection	No software was used for data collection.
Data analysis	Data analysis was performed using code written in the freely available R programming language (R version 3.6.3 (2020-02-29)), and is found as an R package on https://github.com/LizaDarrous/lhcMR. We also used several R-packages for data analysis: 'TwoSampleMR' (version 0.5.5, freely available from https://github.com/MRCIEU/TwoSampleMR), 'GenomicSEM' (version 0.0.2, freely available from https://github.com/GenomicSEM/GenomicSEM) 'cause' (version 1.2.0.314, freely available from https://github.com/jean997/cause), 'epigraphdb' (version 0.2.1, freely available from https://github.com/MRCIEU/epigraphdb-r).

For manuscripts utilizing custom algorithms or software that are central to the research but not yet described in published literature, software must be made available to editors and reviewers. We strongly encourage code deposition in a community repository (e.g. GitHub). See the Nature Portfolio guidelines for submitting code & software for further information.

Data

Policy information about availability of data

All manuscripts must include a data availability statement. This statement should provide the following information, where applicable:

- Accession codes, unique identifiers, or web links for publicly available datasets
- A description of any restrictions on data availability
- For clinical datasets or third party data, please ensure that the statement adheres to our policy

The origin and unique identifier of each of the summary statistics data used is referenced in Supplementary Table 1.
The UK Biobank summary statistics data used in this study can be downloaded from http://www.nealelab.is/uk-biobank . Data on coronary artery disease\cite {CADdata} have been contributed by the CARDIoGRAMplusC4D and UK Biobank CardioMetabolic Consortium CHD working group who used the UK Biobank

Resource (application number 9922). Data have been downloaded from http://www.cardiogramplusc4d.org/data-downloads. Furthermore, we used EpiGraphDB, an analytical platform and database to support data mining in epidemiology, to preform Phenome-wide MR search. Access to EpigraphDB is free and may be done through their web application (https://epigraphdb.org) or their R package (https://github.com/MRCIEU/epigraphdb-r).
The computed local LD scores described in Supplementary Methods 1.3 can be downloaded from https://wp.unil.ch/sgg/lhc-mr/

Field-specific reporting

Please select the one below that is the best fit for your research. If you are not sure, read the appropriate sections before making your selection.

- [x] Life sciences
- [ ] Behavioural & social sciences
- [ ] Ecological, evolutionary & environmental sciences

For a reference copy of the document with all sections, see nature.com/documents/nr-reporting-summary-flat.pdf

Life sciences study design

All studies must disclose on these points even when the disclosure is negative.

Sample size	Sample sizes were reported in GWAS summary statistics and used as needed in our analysis. Effective sample size for case-control GWAS summary statistics was calculated using N_eff = (4*Ncase*Ncontrol)/(Ncase + Ncontrol).
Data exclusions	SNP data for those falling in the HLA region of chromosome 6 were excluded due to the abundance of SNPs associated with autoimmune and infectious diseases as well as the complicated LD structure present in that region. This exclusion criteria was pre-established, and is commonly done in GWAS analysis studies. Otherwise no data was excluded.
Replication	Simulations were replicated 50 times using different data generations for each scenario that was tested. As for real data/traits analysis, no sufficiently large other data set was available for replication. However, the results are fully reproducible with the code available (https://github.com/LizaDarrous/lhcMR).
Randomization	There was no group assignment performed in this study, since it was not experimental.
Blinding	Blinding is not relevant to our study since it was not experimental, and all data collection and processing was done in previous studies.

Reporting for specific materials, systems and methods

We require information from authors about some types of materials, experimental systems and methods used in many studies. Here, indicate whether each material, system or method listed is relevant to your study. If you are not sure if a list item applies to your research, read the appropriate section before selecting a response.

Materials & experimental systems

n/a	Involved in the study	
☒	☐	Antibodies
☒	☐	Eukaryotic cell lines
☒	☐	Palaeontology and archaeology
☒	☐	Animals and other organisms
☒	☐	Human research participants
☒	☐	Clinical data
☒	☐	Dual use research of concern

Methods

n/a	Involved in the study	
☒	☐	ChIP-seq
☒	☐	Flow cytometry
☒	☐	MRI-based neuroimaging
